# REPIN1 regulates iron metabolism and osteoblast apoptosis in osteoporosis

**DOI:** 10.1038/s41419-023-06160-w

**Published:** 2023-09-25

**Authors:** Yu Xia, Gaoran Ge, Haixiang Xiao, Mingzhou Wu, Tianhao Wang, Chengyong Gu, Huilin Yang, Dechun Geng

**Affiliations:** 1 Department of Orthopedics, The First Affiliated Hospital of Soochow University, Suzhou, Jiangsu China; 2 Taicang TCM Hospital Affiliated to Nanjing University of Chinese Medicine, Taicang, China; 3 Suzhou Hospital of Nanjing Medical University, Suzhou Municipal Hospital (North District), Suzhou, China

**Keywords:** Mechanisms of disease, Osteoporosis

## Abstract

Osteoporosis is not well treated due to the difficulty of finding commonalities between the various types of it. Iron homeostasis is a vital component in supporting biochemical functions, and iron overload is recognized as a common risk factor for osteoporosis. In this research, we found that there is indeed evidence of iron accumulation in the bone tissue of patients with osteoporosis and REPIN1, as an origin specific DNA binding protein, may play a key role in this process. We revealed that sh-*Repin1* therapy can rescue bone loss in an iron-overload-induced osteoporosis mouse model. Knockdown of *Repin1* can inhibit apoptosis and enhance the resistance of osteoblasts to iron overload toxicity. REPIN1 promoted apoptosis by regulating iron metabolism in osteoblasts. Mechanistically, knockdown of *Repin1* decreased the expression of *Lcn2*, which ameliorated the toxic effects of intracellular iron overload. The anti-iron effect of lentivirus sh-*Repin1* was partially reversed or replicated by changing LCN2 expression level via si-RNA or plasmid, which indirectly verified the key regulatory role of LCN2 as a downstream target. Furthermore, the levels of BCL2 and BAX, which play a key role in the mitochondrial apoptosis pathway, were affected. In summary, based on the results of clinical specimens, animal models and in vitro experiments, for the first time, we proved the key role of REPIN1 in iron metabolism-related osteoporosis.

## Introduction

As a worldwide public health problem, osteoporosis is mainly manifested by the increased risk of fracture and damage of bone microstructure. Osteoporosis is not well treated due to the difficulty of finding commonalities between the various types of it [1]. Finding a common risk factor for all types of osteoporosis has become an urgent problem to be solved. Several clinical studies have shown that osteoporosis is a common complication of those diseases that will lead to chronic iron accumulation in body [2, 3] and seems to be a result of iron overload. Decreased BMD (Spine BMD Z score < −1SD) is present in 55.1% of children (6–10 years), 72.3% of adolescents (11–19 years) and 87.4% of adults ( > 20 years) with thalassemia. A very high prevalence (72%) of low BMD in adults (18–51 years) with sickle cell disease (SCD) was documented in previous studies [4]. Another cross-sectional study suggested that approximately 70% of SCD patients with abnormally high serum iron levels have osteoporosis, suggesting that iron overload indeed impaired bone formation and induced osteoporosis [5]. Approximately 25–34% of hereditary hemochromatosis (HH) patients show symptoms of osteoporosis, and many more of them are characterized by loss of bone mass [6, 7]. The progression of osteoporosis is often closely related to the severity of iron overload [8], and this phenomenon has also been reported in patients with primary osteoporosis [9, 10]. Tsay et al. successfully created an iron-overload-induced osteoporosis model in mice by injecting iron dextran [11]. Jia et al and Kudo et al obtained the same results using a similar method [12, 13] Iron overload has been repeatedly reported to be associated with inhibiting osteoblast activity and inducing apoptosis [14–16]. Overall, iron overload is a new independent risk factor for several types of osteoporosis and deserves further study.

As a highly reactive metal, Iron is recognized as an essential metallic substance in various biological metabolic processes like cell apoptosis, host defense, mitochondrial respiration, catalyzing the formation of free radicals by inducing Fenton reactions and being involved in the regulation of cell proliferation [17–19]. In human body, the iron pool is made up of the iron in hemoglobin (2 g), plasma(4 mg), macrophages(600 mg), tissue mainly including bone marrow(300 mg), liver(1 g), dietary absorption(1 ~ 2 mg/d) and unregulated loss primarily through epithelial desquamation or blood loss(−1 ~ 2 mg/d) which is in dynamic equilibrium [20]. Iron homeostasis is a vital component in supporting biochemical functions, and consequently, iron overload is presently recognized as a risk factor for infection, cancer, endocrinopathy, cardiomyopathy, neurodegenerative and bone diseases [21–25]. Patients with thalassemia, SCD and HH who show more signs of iron overload in multiple organs frequently have serious osteoporosis [4, 26–28]. Although much evidence suggests that iron overload is associated with osteoporosis, the specific mechanism is still not well explained. The exploration of the deep mechanism of iron metabolism may provide new insight for osteoporosis treatment.

Inhibited osteoblast proliferation and differentiation along with increased amount of adipose tissue are observed in bone marrow cavity of patients with osteoporosis [29], while excess iron also impairs osteoblast function and impacts adipose tissue in association with insulin resistance [10, 30, 31]. In our research, we found that replication initiator 1 (REPIN1) was highly expressed in bone tissue from elderly osteoporosis patients with iron overload. REPIN1 is a zinc finger protein with a molecular weight of 60kda consisting of three clusters. The gene maps to mouse chromosome 6, rat chromosome 4 and human chromosome 7 [32]. REPIN1 has been considered to be an origination-specific DNA binding protein due to its enhanced role in DNA replication [33, 34]. The function of REPIN1 has not been thoroughly studied since discovered, but the results of existing studies suggest that *Repin1* may be worthy of study as a candidate gene in the following processes: adipogenesis, glucose and fatty acid transport, lipid droplet formation and fusion, which plays a regulatory role in fat formation [35, 36] Whether REPIN1 can affect the occurrence of osteoporosis by regulating iron metabolism has not been reported.

## Result

### REPIN1 increased in osteoporosis patients and regulated iron metabolism in vitro

**Fig. 1:**
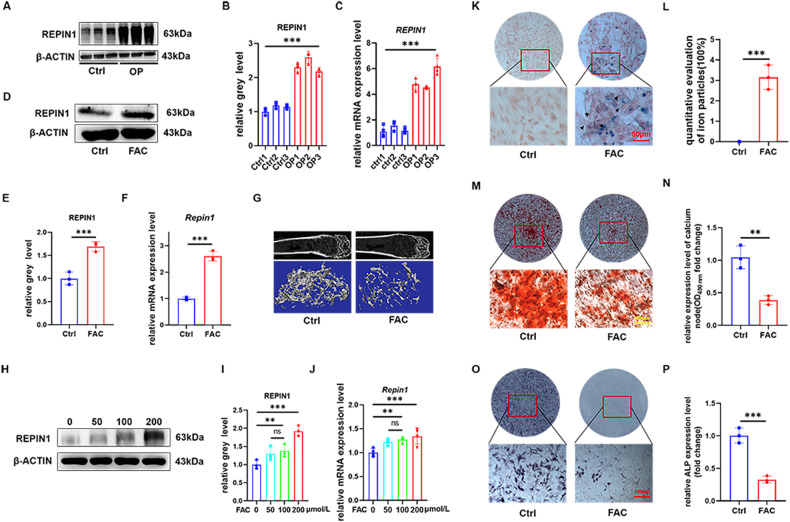
The expression of REPIN1 increases in osteoporosis patients, and iron overload impairs osteogenic function in vitro. **A, B** The expression levels of REPIN1 in bone tissues from osteoporosis patients were analyzed by western blotting. **C** The mRNA levels of *REPIN1* from osteoporosis patients were analyzed. **D, E** The expression level of REPIN1 in bone tissues of mice was analyzed by western blotting. **F** The mRNA levels of *Repin1* were analyzed by qRT‒PCR. **G** Representative micro-CT reconstruction images of trabecular bone under the distal femur growth plate in the control and FAC groups. **H, I** The expression levels of REPIN1 in BMSCs exposed to FAC (0–200 μmol/L) were analyzed by western blotting. **J** The mRNA levels of *Repin1* in BMSCs exposed to FAC (0–200 μmol/L) were analyzed by qRT‒PCR. **K** Perl’s Prussian blue staining was used to stain the iron particles in cells. The blue-stained iron particles are indicated by arrows. **L** Semiquantitative evaluation of iron particles after Perl’s Prussian blue staining. **M** ARS staining at 21 days. **N** Semiquantitative evaluation of the ARS recovery ratio (fold change). **O** ALP staining at 7 days. **P** Relative ALP expression level (fold change). (Cells in **G**–**J** were exposed to FAC at concentrations of 200 μmol/L for 48 h before examination. Values are shown as the means ± SDs **p < 0.01 and ***p < 0.005, *n* = 3 per group all these studies were performed at least three biological replicates).

We compared the gene expression of normal elderly with elderly osteoporosis patients and found that the expression of *REPIN1* increased in the osteoporosis group (log FC = 6.33). Analyses were conducted on the raw data acquired from the Gene Expression Omnibus (GEO, https://www.ncbi.nlm.nih.gov/geo) database (GSE35958) (Fig. S1A). To verify the results of GEO data analysis, we collected bone tissue samples from clinical osteoporosis patients to detect the expression level of REPIN1. We found that the expression level of REPIN1 was indeed increased in osteoporosis patients (Fig. 1A–C). The patients’ demographic data and bone metabolism indicators can be seen in Fig. S1B–F, and there is indeed evidence of iron accumulation in the bone tissue of patients with osteoporosis (Fig. S1G). According to preliminary experiments in animal models, we also found that iron-overload-induced osteoporosis in mice (Fig. 1G). Western blotting and qRT‒PCR analyses showed that the expression of REPIN1 was increased in mice bone tissues (Fig. 1D–F). Western blotting and qRT‒PCR analyses suggested that BMSCs from iron overload mice showed reduced osteogenic ability (Fig. S2A–I). In vitro, BMSCs were exposed to FAC (0–3200 μmol/L) for 48 h, and the CCK-8 results showed that FAC decreased cell viability by approximately 40% at concentrations of 200 μmol/L, and the effect was dose-dependent (Fig. S1H). Therefore, we chose an appropriate concentration as the experimental condition (200 μmol/L). To further determine whether REPIN1 expression is altered in iron-induced osteoporosis, western blotting and quantitative analysis proved that the protein levels of REPIN1 were significantly higher in BMSCs exposed to FAC in a dose-dependent manner (Fig. 1H, I). The qRT‒PCR analysis was consistent with the western blotting results (Fig. 1J). Furthermore, we observed that the cells showed the brown color of FAC after centrifugation (Fig. S2J). The results of Perl’s Prussian blue staining suggested an increase in iron content in BMSCs treated with FAC (Fig. 1K, L), which was similar to the FerroOrange test (Fig. S2K, L). The effect of iron overload on the osteoblastic ability of BMSCs was also confirmed by ALP and ARS results (Fig. 1M–P). These results suggest that iron overload Inhibited BMSCs activity and REPIN1 may be related to iron-induced osteoporosis.

### *Repin1* knockdown mitigated iron-induced suppression of osteogenesis and mineralization

To gain insight into the efficacy of *Repin1* during iron metabolism in osteoblasts, sh-*Repin1* lentivirus particles were used to suppress *Repin1* expression. We obtained 3 sh-*Repin1* lentiviruses (LV-697, LV-1158, LV-1254), and their knockdown effect was confirmed by fluorescence microscopy, western blotting and RT‒qPCR (Fig. S3A–D). LV-697, as most effective sh-*Repin1* lentivirus (The fluorescence intensity of GFP is greater than 90%, the protein expression is knocked down to less than 50%, and the gene expression is knocked down to less than 30%), was chosen for subsequent experiments. To verify the effect of lentivirus, we transfected MC 3T3 E1 cells with sh-*Repin1* (LV-697) again (Fig. S3E, F, H). The infectious efficiency tested by western blotting and RT‒qPCR showed that the expression levels of REPIN1 were significantly reduced.

**Fig. 2:**
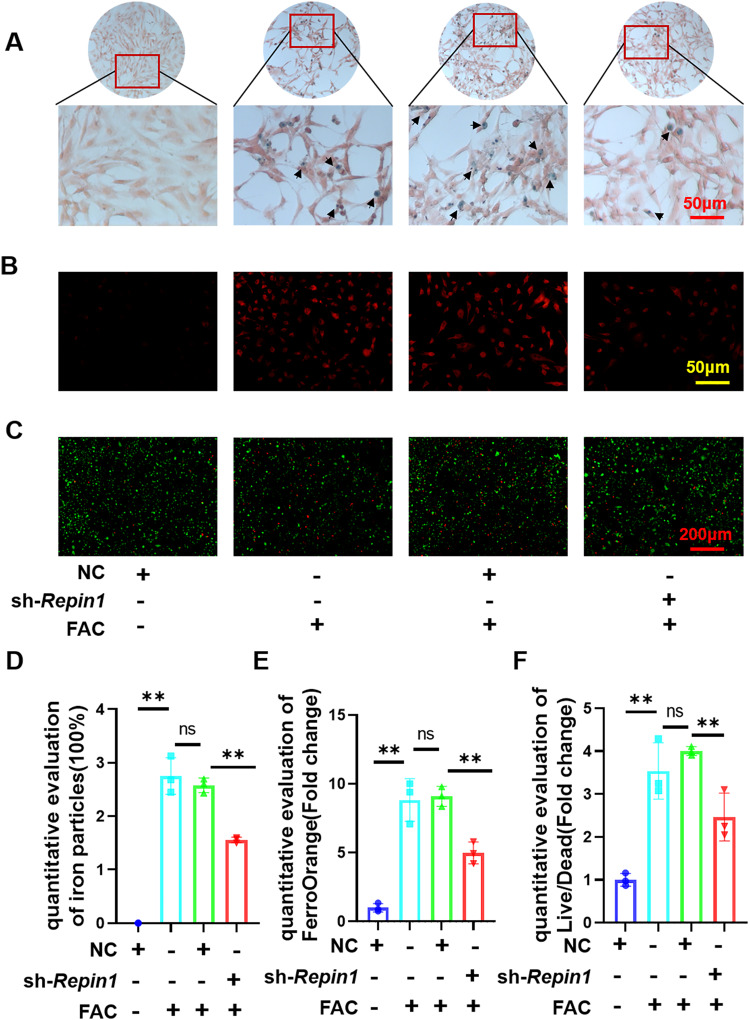
*Repin1* knockdown alters iron accumulation and enhances resistance to iron toxicity in MC 3T3 E1 cells. **A** Perl’s Prussian blue staining was used to stain the iron particles in cells. The blue-stained iron particles are indicated by arrows. **B** FerroOrange fluorescent probe was used to measure intracellular iron content. **C** Cell viability was observed by live/dead staining. Cells in **A**–**C** were exposed to FAC at concentrations of 200 μmol/L for 48 h before examination. **D** Semiquantitative evaluation of iron particles after Perl’s Prussian blue staining. **E** Quantitative evaluation of FerroOrange (Fold change). **F** Quantitative evaluation of Live/Dead. (Values are shown as the means ± SDs. **p < 0.01, *n* = 3 per group, all studies were performed with at least three biological replicates).

In order to observe the effect of REPIN1 on iron metabolism, Perl’s Prussian blue staining was used to stain the iron in cells after treatment with 200 μmol/L FAC for 48 h, and as expected, the iron particles were obviously reduced in the sh-*Repin1* group (Fig. 2A, D). The results of FerroOrange also agree with the Perl’s Prussian blue staining (Fig. 2B, E). Cell viability was observed by live/dead staining, and the results showed that FAC increased the number of dead cells and, to a certain extent, sh-*Repin1* treatment counteracted the iron toxicity provided by FAC (Fig. 2C, F). These results confirmed that knocking down *Repin1* could reduce iron accumulation and increase cell viability after FAC treatment.

**Fig. 3:**
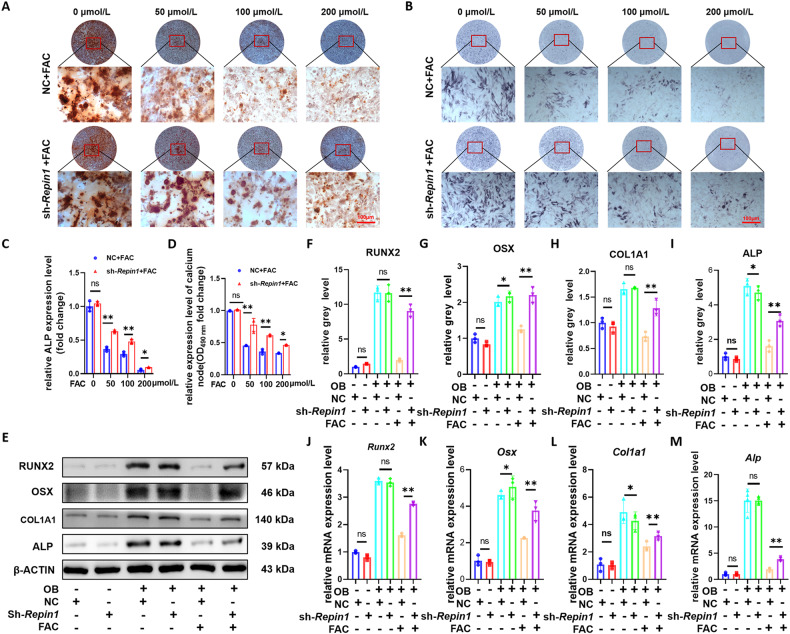
*Repin1* knockdown mitigated iron-overload-induced suppression of osteogenesis and mineralization. **A** Representative images of ARS staining at 21 days. **B** Representative images of ALP staining at 7 days. **C** Relative ALP expression level (fold change). **D** Semiquantitative evaluation of the ARS recovery ratio (fold change). Cells after lentivirus transfection were exposed to FAC at various concentrations (0–200 μmol/L) during osteogenic induction. **E** The expression levels of RUNX2, OSX, COL1A1 and ALP in MC 3T3 E1 cells were analyzed by western blotting after 4 d of osteogenic induction in the presence of 200 μmol/L FAC. **F**–**I** Quantitative analysis of western blotting. **J**–**M** The mRNA levels of *Runx2, Osx, Col1a1 and Alp* were analyzed. (Values are shown as the means ± SDs. *p < 0.05, **p < 0.01 and ***p < 0.005, *n* = 3 per group, all studies were performed with at least three biological replicates).

To test iron damage to osteogenesis and whether sh-*Repin1* treatment could enhance resistance to iron toxicity, we added different concentrations of FAC (0–200 μmol/L) into osteogenic differentiation medium. When the cells were cultured for 7 and 21 days, ALP and ARS showed that treatment with sh-*Repin1* moderated the inhibition of FAC-induced osteogenic differentiation of MC 3T3 E1 cells. Quantitative analysis of ARS and ALP indicated that the mineralization of cells was greatly accelerated in the sh-*Repin1* + FAC group relative to the NC + FAC group (Fig. 3A–D).

Additionally, the results of western blotting and quantitative analysis showed that the expression of osteogenic marker proteins such as RUNX2, ALP, COL1A1 and OSX was decreased after FAC intervention and rescued by sh-*Repin1* treatment (Fig. 3E–I). Furthermore, the western blotting results were confirmed by qRT‒PCR analysis (Fig. 3J–M). These results suggest that the reduction in osteoblast capacity caused by iron overload can be reversed by knocking down *Repin1*.

### *Repin1* knockdown alters the gene expression profiles of osteoblasts and reduces apoptosis by downregulating *Lcn2* expression

**Fig. 4:**
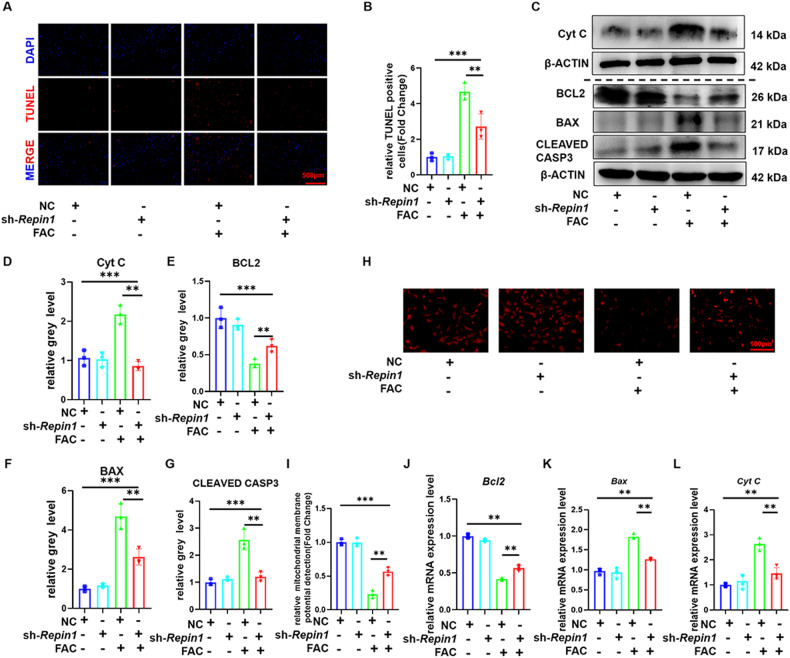
*Repin1* regulates iron-overload-induced osteoblast apoptosis. **A** TUNEL staining of MC 3T3 E1 cells exposed to 200 μmol/L FAC for 48 h. **B** Relative TUNEL-positive cells (Fold Change). **C** The expression levels of Cyt C, BCL2, BAX and CLEAVED CASP3 were analyzed by western blotting. **D**–**G** Quantitative analysis of western blotting. **H, I** Mitochondrial membrane potential detection. **J**–**L** The mRNA levels of *Cyt c, Bcl2* and *Bax* were analyzed by qRT‒PCR. (Values are shown as the means ± SDs **p < 0.01 and ***p < 0.005, *n* = 3 per group, all studies were performed with at least three biological replicates).

Previous studies have shown that apoptosis pathways play an important role in iron-overload-induced osteoporosis. Similarly, TUNEL staining also showed that iron overload caused increased TUNEL-positive cells in BMSCs (Fig. S4A, C). Meanwhile, the protein level of BCL2 was decreased while Cyt C, BAX and CLEAVED CASP3 were increased in the FAC group compared with the Ctrl group (Fig. S4B, D–G). This result was verified by qRT‒PCR (Fig. S4H–J). To further verify whether *Repin1* affects the iron metabolism of osteoblasts through apoptosis, TUNEL staining results showed that the number of TUNEL-positive cells increased in NC + FAC group and decreased in sh-*Repin1* + FAC group (Fig. 4A, B). Western blotting confirmed that the protein levels of Cyt C, BAX and CLEAVED CASPASE3 were increased while BCL2 was downregulated in the NC group treated with FAC. At the same time, sh-*Repin1* treatment reversed the effect of iron overload on protein expression (Fig. 4C–G). This result was verified by qRT‒PCR (Fig. 4J–L). As a hallmark of apoptosis, mitochondrial membrane potential also decreased with FAC treatment and was rescued by sh-*Repin1* therapy (Fig. 4H, I).

**Fig. 5:**
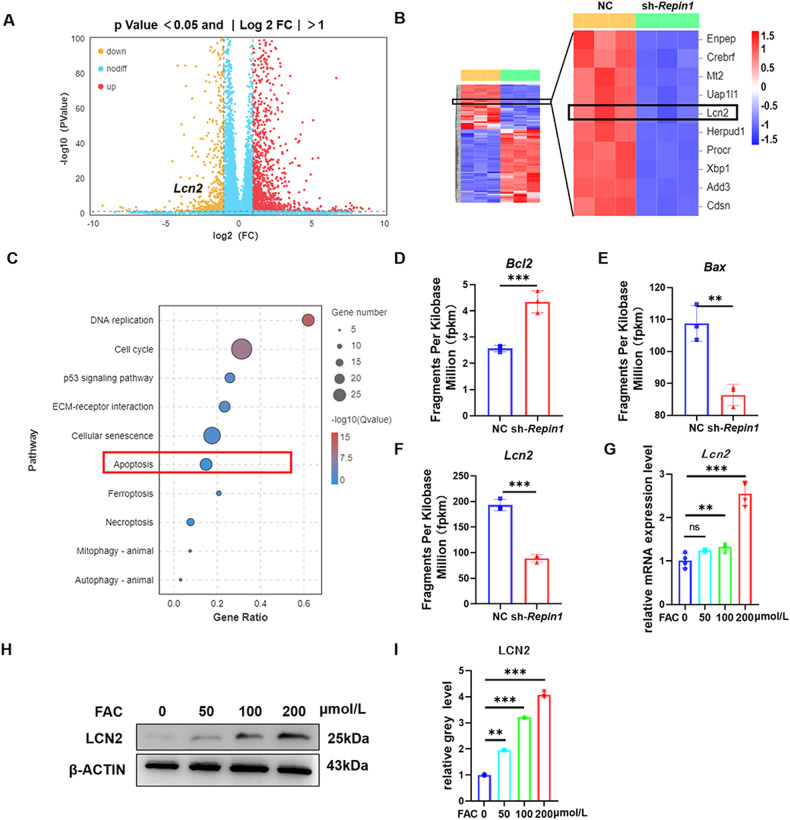
*Repin1* knockdown alters the gene expression profiles of MC 3T3 E1 cells and resists iron overload via the apoptosis pathway. **A** Volcano plots of all differentially expressed genes ( > 2-fold) in NC- and sh-*Repin1*-transfected MC 3T3 E1 cells after exposure to 200 μmol/L FAC for 48 h. **B** Heatmaps of all differentially expressed genes ( > 2-fold) in NC- and sh-*Repin1-*transfected MC 3T3 E1 cells after exposure to 200 μmol/L FAC for 48 h. Red represents higher expression, and blue represents lower expression. **C** The enriched KEGG pathways. **D**–**F** The expression of *Lcn2, Bcl2* and *Bax* in sequencing results. **G** The mRNA levels of *Lcn2* were analyzed by qRT‒PCR. **H** The expression levels of LCN2 were analyzed by western blotting. **I** Quantitative analysis of western blotting. Cells in **H, I** were exposed to FAC at various concentrations (0–200 μmol/L) for 48 h before examination (values are shown as the means ± SDs **p < 0.01 and ***p < 0.005, *n* = 3 per group, all studies were performed with at least three biological replicates).

*Repin1*-knockdown MC 3T3 E1 cells were cultured in 200 μmol/L FAC for 48 h and sent for RNA sequencing (RNA-seq) to observe the transcriptome changes. We aimed to explore the undetected molecular mechanisms by which *Repin1* regulates osteoblast differentiation during iron overload condition. In total, 1739 genes with significantly differential expression (1213 up, 526 down) in *Repin1*-knockdown cells compared to control were identified ( | fold change | > 2, *P* < 0.05, and FDR < 0.05) (Fig. 5A, B). The enriched KEGG pathways suggested that apoptosis might play a decisive role in this process (Fig. 5C), and the RNA-seq results revealed that the expression levels of the functional apoptosis genes *Bcl2* and *Bax* changed significantly (Fig. 5D, E). Excitingly, our analysis identified a gene that may play a key role in iron-overload-induced osteoblast apoptosis. We observed that the expression of *Lipocalin2* (*Lcn2*) which is considered to play an important role in regulating cell iron levels in both physiological and inflammatory conditions, was dramatically decreased in the sh-*Repin1* group (Fig. 5F), the RNA-seq results were further validated by western blotting and RT‒qPCR (Fig. 5G–I).

In combination with previous literature reports that LCN2 is a protein with significant changes after low expression of REPIN1, and considering the important functions of LCN2 in iron metabolism and apoptosis-related pathways, we hypothesize that *Repin1* affects iron metabolism in osteoblasts through *Lcn2* and further affects osteoblast apoptosis induced by iron overload. Western blotting and qRT‒PCR showed decreased expression of LCN2 in the sh*-Repin1* groups, which confirmed our hypothesis (Fig. S5A–C). To provide more evidence, we detected LCN2 protein and RNA expression in clinical specimens and found that the expression of LCN2 was increased in osteoporosis patients, which was mutually supportive of the RNA-seq results (Fig. S5D–F). These results preliminarily demonstrate that REPIN1 regulates iron metabolism and apoptosis through LCN2.

### Altering LCN2 expression reversed or duplicated the therapeutic effect of sh-*Repin1*

**Fig. 6:**
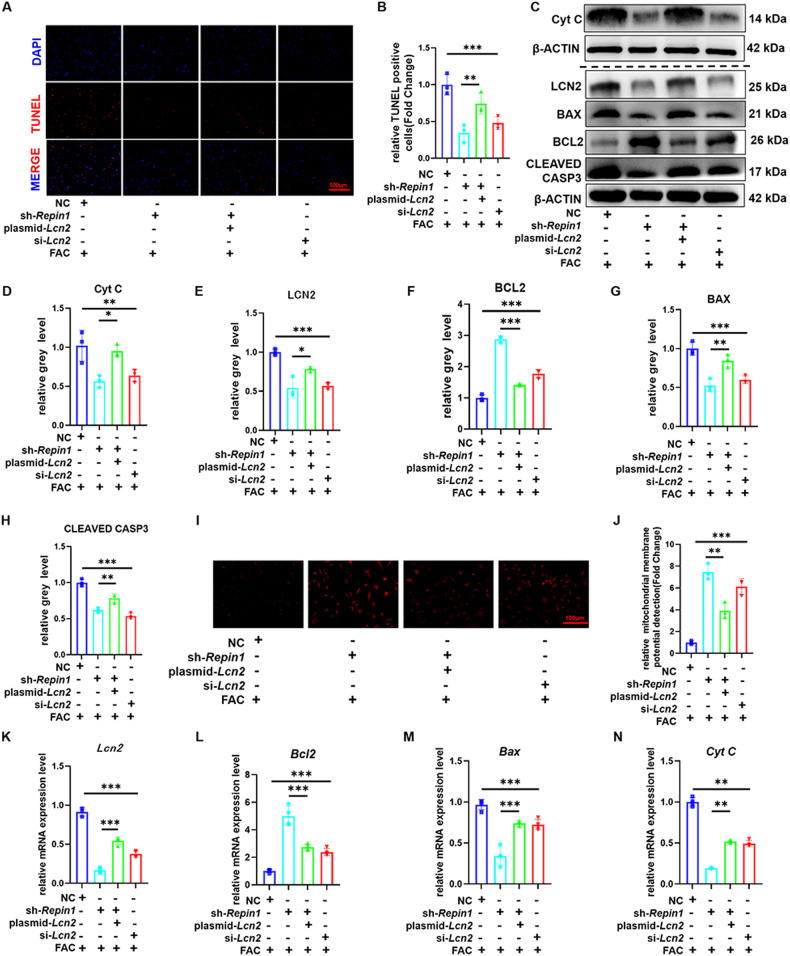
Altering the expression of *Lcn2* can reverse or mimic the effect of sh-*Repin1* on iron-overload-induced osteoblast apoptosis. **A** TUNEL staining of MC 3T3 E1 cells exposed to 200 μmol/L FAC for 48 h. **B** Relative TUNEL-positive cells (Fold Change). **C** The expression levels of Cyt C, LCN2, BCL2, BAX and CLEAVED CASP3 were analyzed by western blotting. **D**–**H** Quantitative analysis of western blotting. **I, J** Mitochondrial membrane potential detection. **K**–**N** The mRNA levels of *Cyt c, Lcn2, Bcl2* and *Bax* were analyzed by qRT‒PCR. (Values are shown as the means ± SDs *p < 0.05, **p < 0.01 and ***p < 0.005, *n* = 3 per grou*p*, all studies were performed with at least three biological replicates).

To further verify the effect of LCN2 on iron-overload-induced osteoblast apoptosis regulated by the REPIN1 pathway, plasmid-*Lcn2* was used to increase the expression of *Lcn2* in sh-*Repin1* and control cells. LCN2 was successfully overexpressed by plasmid-*L*cn2, and the results of fluorescence microscopy, western blotting and qRT‒PCR confirmed the effect (Fig. S6A–C, E–H). siRNA was used to decrease LCN2 expression in cells, and the effect was confirmed by fluorescence microscopy, western blotting and qRT‒PCR (Fig. S6D, I–K). The results of TUNEL staining indicated that the rescue effect of knocking down *Repin1* on apoptosis was antagonized by plasmid-*Lcn2* and mimicked by si-*Lcn2* (Fig. 6A, B). Western blotting confirmed that the protein levels of Cyt C, BAX and CLEAVED CASPASE3 were increased, while BCL2 was downregulated in the plasmid-*Lcn2* group compared with the sh-*Repin1* group. The protein expression level of the si-*Lcn2* group was similar to that of the sh-*Repin1* group (Fig. 6C–H). The western blotting results were consistent with the qRT‒PCR results (Fig. 6K–N). The changes in mitochondrial membrane potential were also consistent with the TUNEL results (Fig. 6I, J). These results suggested that the decreased osteoblast apoptosis induced by iron overload after knockdown of *Repin1* was offset by treatment with overexpression of *Lcn2*. On the other hand, cells with *Lcn2* knockdown by siRNA also had a slightly lower ability to resist apoptosis caused by iron overload than cells with *Repin1* knockdown. Perl’s Prussian blue staining and FerroOrange staining also confirmed that altering LCN2 expression could partially reverse or replicate the regulatory effect of REPIN1 on iron metabolism in iron-overloaded osteoblasts (Fig. S7A–D). Moreover, western blotting showed that the protein levels of RUNX2, OSX, COL1A1 and ALP in the plasmid-*Lcn2* group were lower than those in the sh-*Repin1* group. Meanwhile, the expression of osteogenic marker protein in the si-*Lcn2* group was similar to that in the sh-*Repin1* group (Fig. S7E–I). The western blotting results were revalidated by qRT‒PCR (Fig. S7J–M). The above conclusions proved that the therapeutic effect of REPIN1 on the weakened osteoblast ability induced by iron overload was achieved by altering the expression of LCN2.

### REPIN1 knockdown ameliorates bone loss in osteoporosis mice

**Fig. 7:**
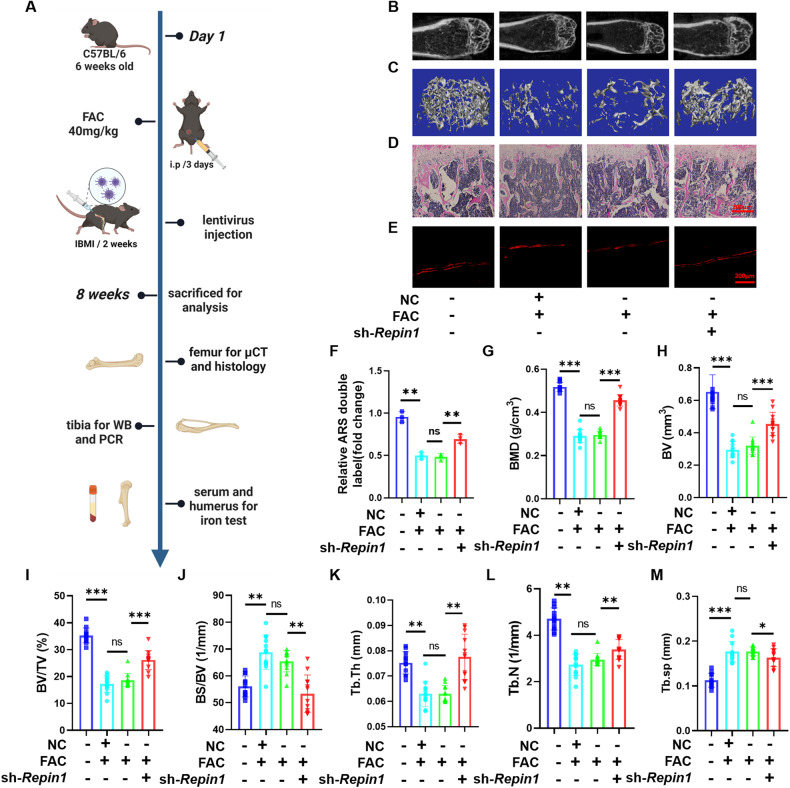
*Repin1* knockdown prevents iron-overload-induced bone loss in vivo. **A** Schematic diagram showing the in vivo experimental design. **B, C** Representative micro-CT reconstruction images of trabecular bone under the distal femur growth plate in the control group, NC + FAC group, FAC group, and sh-*Repin1* + FAC group are shown. **D** Representative H&E staining images of trabecular bone under the distal femur growth plate from the control group, NC + FAC group, FAC group, and Sh-repin1+FAC group are shown. **E** Alizarin red double-label experiment. **F** Quantitative analysis of the width of the alizarin red double label. **G**–**M** Quantitative analysis of bone parameters. The region of interest selected for trabecular analysis started 100 sections below the proximal end of the distal femur growth plate, and 150 slices (6 µm each) were read per sample. **G** BMD (g/cm^3^). **H** BV (mm^3^). **I** BV/TV (%). **J** BS/BV (1/mm). **K** Tb. Th (mm). **L** Tb. N (1/mm). **M** Tb.sp (mm). (Values are shown as the means ± SDs. *p < 0.05, **p < 0.01 and ***p < 0.005, *n* = 6 biologically independent mice per group).

To confirm our previous findings in vitro and clarify the specific mechanistic role of REPIN1 in iron-overload-induced osteoporosis, a sh-*Repin1* lentivirus was used to knockdown REPIN1 expression in iron overload mice (Fig. 7A). Western blotting and qRT‒PCR were used to verify the efficiency of the lentivirus, and the results showed that REPIN1 expression was significantly decreased in collected tibia tissues of mice from the sh-*Repin1* group (Fig. S8A–C). At the same time, we also found similar expression changes in REPIN1 in the OVX model (Fig. S8D–F). The results of Micro-CT analysis and 3D reconstruction of the image showed that the bone mass in the FAC and NC + FAC groups was reduced, and interestingly, the loss was partially relieved after sh-*Repin1* lentivirus treatment (Fig. 7B, C). Similar results were also confirmed in OVX-induced osteoporosis mice (Fig. S9A).

H&E staining was used to observe the morphology of femur tissue in each group to further evaluate the change of bone trabecular micromorphology. Compared with the NC + FAC or FAC-treated group, thinning of bone trabeculae was significantly improved in the sh-*Repin1*-treated group in H&E-stained tissue samples (Fig. 7D). In addition, an Alizarin red double-label experiment was used to test the rate of bone formation, and the results showed that the distance between the two labels was significantly shortened within the FAC and NC + FAC groups, while the distance was wider in the sh-*Repin1* treatment group (Fig. 7E, F). Quantitative analysis of Micro-CT results indicated that compared with control mice received intraperitoneal saline injections, BMD, BV, BV/TV, Tb.th, Tb. N of mice received FAC were decreased, while BS/BV and Tb.sp were increased. These changes were reversed when *Repin1 was knocked down* (Fig. 7G–M), and similar results were confirmed in OVX-induced osteoporosis mice (Fig. S9B–F). In summary, we found the therapeutic effect of sh-*Repin1* in saving bone mass in osteoporosis models induced by iron overload and OVX, which fully proved that *Repin1* plays a universal role in various types of osteoporosis.

In order to further verify the important role of *Repin1* in iron metabolism in vivo. Perl’s Prussian blue staining was used in mouse femur tissue sections to evaluate the iron in bone tissue, and the results showed that a large number of blue-stained iron particles were observed in the bone marrow cavity of the FAC and NC + FAC groups, while this phenomenon was significantly reduced in mice treated with sh-*Repin1* (Fig. S10A). FAC also increased the serum iron concentration (Fig. S10D). TUNEL staining of tissue sections showed increased apoptosis in the FAC and NC + FAC groups compared with the control group, as sh-*Repin1* treatment alleviated the occurrence of apoptosis in vivo (Fig. S10B, C). These results suggest that REPIN1 alters iron metabolism and apoptosis in osteoporosis mice.

## Discussion

Osteoporosis has long been undertreated because it is difficult to identify a common risk factor for all types of osteoporosis and to target it with interventions. In recent years, osteoporosis has often been reported as a common complication of these diseases, which manifests as iron accumulation in vivo [17, 26, 27, 37], including hereditary diseases such as hereditary hemochromatosis, thalassemia, atransferrinemia and sickle cell anemia and acquired diseases such as chronic liver diseases and hemochromatosis [38–40]. On the other hand, some clinical studies have also found that primary osteoporosis is accompanied by iron overload [10, 41]. As a public health problem endangering global health, osteoporosis and iron overload appear to be cause-and-effect, with either contributing to the other’s progress. While the causal relationship between osteoporosis and iron overload, like the chicken-egg cycle, remains difficult to elucidate, the available evidence suggests that iron overload as an independent risk factor for several types of osteoporosis deserves further investigation. To eliminate unwanted distractions, our research focused on iron overload as the cause and successfully established a mouse model of osteoporosis induced by iron overload that can be improved by sh-*Repin1* treatment. The results indicate that intraperitoneal injection of FAC can effectively construct a mouse model of osteoporosis induced by iron overload. This method is simple and effective, and should be popularized as a routine model for the study of osteoporosis related to iron metabolism. The injection of lentivirus effectively improved the performance of osteoporosis in mice. The results in animal models preliminarily confirmed the conclusion that REPIN1 plays a key regulatory role in iron-overload-induced osteoporosis. This phenomenon has also been verified in the OVX model, indicating that *Repin1*’s regulation of osteoporosis is universal. We observed similar therapeutic effects in the FAC and OVX models, further confirming the key regulatory role of REPIN1, which will be important for the subsequent search for common therapeutic targets for multiple types of osteoporosis. In vitro experiments, we found that iron overload had a dose-dependent toxic effect on osteoblast differentiation, and this process is closely related to apoptosis and iron metabolism. It is worth noting that we combined bioinformatics analysis through in vivo and in vitro experimental verification for the first time and found the key role of REPIN1 in iron metabolism-related osteoporosis. This finding was further elucidated through the apoptosis pathway.

REPIN1 is a zinc finger protein with replication enhancement activity [32, 36], and its cellular function has not been thoroughly understood since its discovery. Previous studies have suggested that REPIN1 may be involved in fat metabolism and insulin resistance [42–45]. Osteoporosis patients often experience a breakdown in the balance of adipose and bone formation [29, 46, 47]. Based on the raw data from the GEO database, we first found a trend of high expression of *Repin1* in osteoporosis patients and then verified this finding in clinical specimens. We found that the expression of REPIN1 was increased and that there was also accumulation of iron in bone tissue from osteoporosis patients. REPIN1 and iron were found to be elevated in osteoporosis, and combined with the current role of iron overload in osteoporosis, our research successfully linked REPIN1 and osteoporosis through iron metabolism-related pathways. We constructed a mouse model of osteoporosis induced by iron overload and confirmed the therapeutic effect of sh-*Repin1*. In vitro experiments, we successfully constructed an iron overload cell model by using FAC and observed the damage of iron overload on the function of osteoblasts, which was accompanied by the accumulation of iron particles in cells and an increase in apoptosis. In particular, according to our RNA-seq results, we found that REPIN1 regulated the iron metabolism of osteoblasts through LCN2, thus affecting the occurrence of apoptosis. In brief, combined with the findings of the GEO database, we repeatedly verified the key role of *Repin1* in iron-overload-induced osteoporosis from multiple aspects through clinical specimens, in vitro and in vivo experiments, and bioinformatics analysis after RNA-seq.

Lipocalin2 (LCN2) is a secreted glycoprotein that belongs to a group of transporters of small lipophilic molecules in circulation which is also known as neutrophil gelatinase-associated lipocalin (NGAL) in humans [48, 49]. It has been reported that LCN2 is the most strongly modified gene after altering REPIN1 expression, which is consistent with our RNA-seq results [44, 45], and plays a key role in the regulation of apoptosis and inflammation, transport of iron, and maintaining normal physiological function [50, 51]. Previous studies have revealed that serum LCN2 was increased in patients who had β‐thalassemia [52, 53] and that LCN2 mRNA and protein levels were higher in obese patients than in lean subjects [54]. Wai H Lim et al. reported that serum levels of LCN2 were associated with the risk of fragility fractures in elderly patients. Timely monitoring of serum LCN2 levels is helpful to predict fracture risk and improve fracture prognosis in the elderly [55]. Costa et al. found that overexpression of LCN2 in transgenic mice bones resulted in reduced osteoblast capacity enhanced osteoclast activity, resulting in decreased bone mass in mice [56]. Meanwhile, another study showed that LCN2 is an osteoblast mechanoresponsive gene which will increase with reduced activity or gravity load [57, 58]. In this research, we found that *Lcn2*, as a target gene of *Repin1*, was involved in the process of osteoblast apoptosis induced by iron overload. The RNA-seq results in this study confirmed our suspicions. We used siRNA and plasmid to knockdown or overexpress *Lcn2*, respectively, which partially offset or replicated the therapeutic effect of sh-*Repin1* in iron-overload-induced osteogenesis dysfunction. This suggests that LCN2, as the downstream target of REPIN1, plays a crucial role in regulating iron metabolism in osteoblasts. Furthermore, the downstream mechanism of LCN2 still has broad exploration space. Two membrane receptors of LCN2 have been widely reported, namely LPR2 (also known as megalin) and 24p3R (also known as SLC22A17) [50, 59]. LPR2 is a polygamous entotic receptor expressed primarily by renal epithelial cells to promote renal reabsorption of LCN2. Barasch et al. found that the use of a patented mutant form of LCN2 can alter the affinity with LPR2 and thus alter the level of circulating iron excretion [60]. 24p3R belongs to the family of organic cationic transporters and is expressed in many tissues. Devireddy et al. found that different states of LCN2 binding to 24p3R have opposite effects on intracellular iron concentrations. 24p3R May therefore be able to activate different signaling pathways, depending on the state of LCN2 [61]. In this study, LCN2 was confirmed to be involved in the process of iron metabolism and cell apoptosis. Combined with the findings in this study and the results of existing literature, we are more inclined to speculate that LCN2 binds to its receptor 24p3R and activates downstream signaling pathways to play a role, thus affecting osteoblast function, but the specific mechanism is still a mystery.

In our research, through enrichment analysis of differentially expressed genes from RNA-seq results, we found that iron overload caused by FAC can induce osteoblast apoptosis, while knockdown of REPIN1 can inhibit the occurrence of apoptosis and enhance the resistance of osteoblasts to iron overload toxicity. Our results for the first time found that this is through regulating the expression of LCN2, thus affecting the levels of Cyt C, BAX and BCL2. These phenomena indicate that the mitochondrial apoptosis pathway plays a key role. The role of mitochondrial pathways in apoptosis has been widely established [62–64], and mitochondrial apoptosis signaling pathways are regulated by antiapoptotic proteins (such as BCL2) and proapoptotic proteins (such as Cyt C, BAX). The decrease in BCL2 regulates apoptosis by increasing mitochondrial permeability. BAX acts against BCL2 and induces the release of cytochrome C, ultimately leading to the activation of CASPASE3 [65, 66]. In this study, we found that iron overload caused an increase in apoptosis, which was achieved by decreasing the expression of BCL2 and increasing the expression of Cyt C, BAX and CLEAVED CASP3. The changes in mitochondrial membrane potential were also consistent with our conjecture. To further verify this, we used plasmid and siRNA to change the expression of *Lcn2* and proved that LCN2 was the key protein involved in the effect of REPIN1 on osteoblast apoptosis. This function may be achieved by altering the relative expression of Cyt C, BAX and BCL2, thus affecting the transition of mitochondrial membrane potential, which is worth further investigation. At the same time, the occurrence of apoptosis also seems to be closely related to the accumulation of Fe^2+^. In this study, FerroOrange was used to detect Fe^2+^ in cells, and its level was positively correlated with apoptosis. This phenomenon is probably due to the accumulation of Fe^2+^ in the process of iron overload leading to the increase of Fenton reaction, which produces more free radicals and further causes the occurrence of apoptosis [67]. The different roles of Fe^3+^ and Fe^2+^ in the process of iron overload will be an interesting research direction.

It is also worth mentioning that most of the iron in the body is found in hemoglobin. Dietary iron is absorbed by divalent metal transporter 1 (DMT1), binds to transferrin via ferroportin (FPN), and is eventually absorbed by hepatocytes, macrophages, and bone marrow cells via transferrin receptor 1 (TfR1). At the cellular level, regulation of the expression of proteins involved in iron metabolism, such as ferritin or TfRs, is coordinated through the iron regulatory proteins (IRPs) or iron-responsive elements (IREs) binding proteins. However, there are still pathways different from the mainstream iron metabolism pathway that has not been discovered [68]. LCN2 can interact with iron by forming ternary complexes with siderophores as cofactors. Siderophores are generally classified according to their functional chemical groups into catecholates, carboxylates, and hydroxamates. LCN2 is primarily capable of sequestering siderophores that are catecholates and some carboxylates, but not hydroxamates [50, 69]. The way LCN2 binds to iron differs from common iron transporters, which may be related to its unique calyx structure, the calyx in LCN2 is shallower, broader, and is large enough to accommodate macromolecular ligands. Finding unknown siderophores that can bind LCN2 will also be an interesting research direction in the future.

In conclusion, we found that there was iron overload in osteoporosis patients, and iron overload also caused osteoporosis in vivo and in vitro. The two are mutually causal. Our results suggest that iron overload inhibits osteoblast differentiation by affecting iron metabolism and apoptosis, a process that can be mitigated by knockdown of REPIN1. REPIN1 regulates the expression of LCN2, thereby affecting Cyt C, BAX and BCL2, changing the mitochondrial membrane potential, and acting through the mitochondrial apoptosis pathway. These findings are based on clinical specimens and in vivo and in vitro experiments. Although we have demonstrated the critical regulatory role of *Repin1* in both iron overload and OVX-induced osteoporosis, we will conduct further studies on other osteoporosis models (such as senile osteoporosis, diabetic osteoporosis, etc.) in the future to find the universality of our conclusions and provide new insight into the general target of osteoporosis treatment.

## Materials and methods

### Cell culture

MC 3T3-E1 cells were obtained from the Cell Bank of the Chinese Academy of Sciences (Shanghai, China). BMSCs were obtained from the femurs and tibias according to the protocol [70]. Briefly, BMSCs were extracted by flushing the femur and tibia cavities of male 4-week-old C57BL/6 mice with α-MEM. The medium was changed after 72 h and the cells were purified by passage. Cells were grown in 55 cm^2^ dishes with α-MEM containing 10% fetal bovine serum (FBS) and penicillin/streptomycin antibiotics under 5% CO_2_ at 37 °C (all from Gibco, California, USA). MC 3T3-E1 and BMSCs from passages 3 to 15 were used in this study. After reaching 70–80% confluence, cells were detached and replated in 55 cm^2^ dishes for passage 1:3 or other plates for further research.

### Lentivirus transfection

To knockdown *Repin1*, the sequence of lentiviruses obtained from GenePharma (Suzhou, China) is shown below: NC: TTCTCCGAACGTGTCACGT, sh-*Repin1*-697: GGGTTTCATCTGCCACCTATG, sh-*Repin1*-1158: GGAAGCGTTTCACCAACAAGC, sh-*Repin1*-1254: GCCACAAACCCAACCTGTTGT. MC 3T3-E1 cells were planted in 6-well plates at a density of 2 × 10^5^ / well. Cells were observed 24 h later and transfection began when the density was between 30–50%. Lentivirus (multiplicity of infection [MOI] = 150) was used for transfection. 3 days after transfection, the medium was changed to α-MEM containing 10% FBS and 5ug/ml puromycin. Since the lentivirus contains anti-puromycin genes, purified lentivirus-transfected cell line would be obtained by this method. 2 days later, we preliminarily judged the transfection efficiency of lentivirus by observing the GFP intensity of cells under a fluorescence microscope (Carl Zeiss, Oberkochen, Germany), then used western blotting and RT‒qPCR to verify the actual REPIN1 knockdown effect again. LV-697 as the most effective sh-*Repin1* lentivirus, was chosen for subsequent experiments.

### LCN2 knockdown and overexpression

siRNA and plasmid targeting *Lcn2* were purchased from GenePharma (Suzhou, China). The sequences of the *Lcn2* siRNA were as follows: siRNA: sense 5′-3′: GCCUCAAGGACGACAACAUTT; anti sense 5′-3′: AUGUUGUCGUCCUUGAGGCTT. Plasmid: forward 5′-3′: GCTTGGTACCGAGCTCGGATCCGCCACCATGGCCCTGAGTGTCATGTGTCTGGGCCTTG; reverse 5′-3′: TGCTGGATATCTGCAGAATTCTCAGTTGTCAATGCATTGGTCGGTGGGGACAGAGAAGA. The full-length cDNA encoding mouse *Lcn2* was amplified, and the recombinant plasmid pcDNA3.1/*Lcn2* was constructed. Briefly, 5 days after transfection with LV-sh-*Repin1*, cells were treated with siRNA or plasmid by using Lipo6000 reagent (Beyotime, Shanghai, China, C0526). Transfection efficiency was determined by fluorescence microscope, western blotting and RT‒qPCR.

### RNA-seq

Cells were treated with 200 μmol/L ferric ammonium citrate (FAC, Sigma‒Aldrich, Missouri, USA) for 48 h after transfected by NC or sh-*Repin1* lentivirus. After total cellular RNA was extracted from the different groups by using TRIzol reagent (Beyotime, Shanghai, China, R0016), Transcriptome sequencing and data analysis were conducted by Gene Denovo Biotechnology Co. (Guangzhou, China). The raw data were expressed by fragments per kilobase of exon model per million mapped fragments (FPKM). We used the R 4.0.3 package for differential gene analysis, *P* < 0.05, |fold change | > 2, and FDR < 0.05 is considered as significant, volcano mapping, heat mapping and gene enrichment analysis were used to visualize the results.

### Cell viability assay

The cytotoxicity of FAC was evaluated by Cell Counting Kit-8 (CCK-8) (Sangon Biotech, Shanghai, China, E606335). Cells were seeded in 96-well plates at a density of 5000 per well, then FAC were added into culture medium for 48 h. After that, cells were washed with phosphate buffered saline (PBS) for 3 times and incubated for 1 h at 37 °C with CCK-8 solution. Finally, the absorbance was observed at 450 nm wavelength using a microplate reader (BioTek, Vermont, USA).

A Calcein-AM/PI Double Stain Kit (Yeasen, Shanghai, China, 40747ES76) was used following the manufacturer’s protocol for live/dead staining. Cells were cultured in 24-well plates at a concentration of 20000 cells per well in α-MEM with 200 μmol/L FAC for 48 h. Then, 250 μL testing solution was added and observed under a fluorescence microscope after incubation for 30 min at 37 °C.

### Mitochondrial membrane potential assay

Mitochondrial membrane potential was measured by a mitochondrial membrane potential assay kit with TMRE according to the manufacturer’s protocol (Beyotime, Shanghai, China, C2001S). Cells after intervention were washed with PBS 3 times, then TMRE working solution was added into each well and incubated at 37 °C for 30 min. Fluorescence microscopy was used to observe the mitochondrial membrane potential.

### TUNEL assay

Apoptosis levels in both cells and tissues were measured using a one-step TUNEL apoptosis assay kit (Beyotime, Shanghai, China, C1089) according to the protocol. Cells were immobilized with 4% paraformaldehyde (Beyotime, Shanghai, China, P0099) for 30 min and incubated with 0.3% Triton X-100 (Beyotime, Shanghai, China, P0096) for 5 min. Paraffin sections were incubated with protease K (Beyotime, Shanghai, China, ST532) at 37 °C for 30 min after dewaxing and rehydrating. The specimen was washed in PBS 3 times and incubated in TUNEL working solution at 37 °C away from light for 1 h. Then, the cells or sections sealed with antifade mounting medium with DAPI (Beyotime, Shanghai, China, P0131). TUNEL-positive cells were observed by using a fluorescence microscope.

### Osteoblast differentiation

MC 3T3 E1 cells or BMSCs were seeded at a density of 30000 and grown in α-MEM containing 10% FBS and 1% P/S. After reaching 70–80% density, the medium was changed for osteogenic differentiation α-MEM containing 10% FBS, 10 mM β-glycerophosphate and 0.5 mM vitamin C (Sigma‒Aldrich, Missouri, USA). Replace the fresh medium and different concentrations of FAC (0–200 μmol/L) every 3 days.

### Western blotting analysis

Proteins from different samples were obtained by using radioimmunoprecipitation assay (RIPA) lysis buffer (NCM, Suzhou, China, WB3100) at 4 °C for 30 min. Then the supernatant was kept after centrifugation (13,000 × *g*, 4 °C) for 30 min (Thermo Fisher Scientific, Massachusetts, USA). A BCA kit (NCM, Suzhou, China, WB6501) was used to measure the protein content of the sample. Proteins (30 µg) were separated and electrophoretic in SDS‒PAGE (Vazyme, Nanjing, China, E303-01) and transferred onto nitrocellulose membranes (Beyotime, Shanghai, China, FFN02). Next, the membrane was blocked with NcmBlot blocking buffer (NCM, Suzhou, China, P30500) and incubated in primary antibody solution, including antibodies against REPIN1 (1:500, 25–036, ProSci, California, USA), LCN2 (1:1000, A3176, ABclonal, Massachusetts, USA), RUNX2 (1:1000, ab23981, Abcam, Cambridge, UK), ALP (1:1000, ab229126, Abcam, Cambridge, UK), OSX (1:1000, ab209484, Abcam, Cambridge, UK), COL1A1 (1:1000, A1352, ABclonal, Massachusetts, USA), Cyt C(1:5000, ab 133504, Abcam, Cambridge, UK), BCL2 (1:1000, ab182858, Abcam, Cambridge, UK), BAX (1:1000, ab32503, Abcam, Cambridge, UK), CLEAVED CASP3 (1:5000, ab214430, Abcam, Cambridge, UK) overnight at 4 °C. β-ACTIN (1:1000, ABclonal, AC006) was used as a loading control. After washing 3 times with Western blotting wash buffer (Beyotime, China, P0023C), the corresponding secondary antibody (1:10000, AS014, ABclonal, Massachusetts, USA) was added at room temperature for 1 h. The protein bands were observed under chemiluminescence imaging system (Bio-Rad, California, USA) by using Enhanced Chemiluminescent (NCM, Suzhou, China, P2300) and quantified by ImageJ (NIH, Maryland, USA).

### Quantitative real-time PCR assay

Total RNA was obtained from different samples by using TRIzol reagent and the concentration of RNA was quantified by a NanoDrop 2000 spectrophotometer (Thermo Fisher Scientific, Massachusetts, USA). Then 1 ug RNA was mixed with HiScript III RT SuperMix for qPCR (Vazyme, Nanjing, China, R323-01) for reverse transcription. A mixture containing 5 µl of ChamQ SYBR Color qPCR Master Mix (Vazyme, Nanjing, China, Q421-02), 0.2 μl of forward and reverse primer, 2.6 µl of nuclease-free ddH_2_O and 2 µl of cDNA were added into 96-well pcr plate. After that, qRT‒PCR program was performed in a thermal cycler (Bio-Rad, California, USA). Samples from each group was performed in triplicate, and the relative expression of mRNA was determined using 2^− ΔΔCq^ method. Primers of target genes are listed in Table S1.

### Alkaline phosphatase staining

MC 3T3 E1 cells or BMSCs were fixed in 4% paraformaldehyde for 30 min at 4 °C after induced by osteogenic medium for 7 days. BCIP/NBT working solution (Beyotime, Shanghai, China, C3206) was added into each well, cells were then incubated away from light for 30 min at 37 °C. ALP activity was detected by an Alkaline Phosphatase quantification Kit (Jiancheng Bioengineering Institute, Nanjing, China, A059) according to the manufacturer’s protocol and shown as the fold change.

### Alizarin red S staining

MC 3T3 E1 cells or BMSCs were fixed in ethanol for 30 min at 4 °C after induced by osteogenic medium for 21 days. Alizarin Red S solution (Beyotime, Shanghai, China, C0148S) was added to the cells for 30 min at 37 °C, and the cells were flushed with distilled water at least 3 times before being photographed. A 5% perchloric acid solution was added into each well to dissolve calcium nodules at 37 °C for 30 min to quantify calcium nodules, the absorbance was measured at 490 nm. The relative expression result is represented by the fold change.

### Iron test

Perl’s Prussian blue staining (Solarbio, Beijing, China, G1424/G1426) was used to observe iron in cells and tissues according to the instructions. After cultured in medium with FAC (200 μmol/L) for 48 h, cells were washed with distilled water at least 3 times to ensure that the iron in the medium was completely washed away. Then fixed in 4% paraformaldehyde for 30 min at 4 °C and prepared Perl’s Stain solution was dropped to completely cover the cells and incubated at 37 °C for 30 min. Rinse in distilled water twice. air dried and viewed under a microscope. For paraffin section staining, the samples were dewaxed in xylene and rehydrated in ethanol, washed with distilled water 3 times for 5 min each time. Then stained with Perl’s Stain solution for 30 min and washed with distilled water 3 times for 5 min each time. Next, the sections were redyed with eosin solution for 15 s, rinsed quickly in distilled water for 3 s, dehydrated, transparent, sealed and photographed under a microscope. In the experiment, it was necessary to wash the sections enough times, and the water used was distilled water, metal instruments was prohibited, so as to avoid the interference of external metal ions and reduce the probability of false positives.

A FerroOrange assay kit (DOJINDO, Kumamoto Ken, Japan, F374) was used to measure the concentration of iron ions in cells. FerroOrange working solution with a concentration of 1 μmol/l was added and incubated in an incubator with 5% CO2 at 37 °C for 30 min. The fluorescence intensity was observed using a fluorescence microscope.

Iron content assay kits (Sloarbo, Beijing, China, BC4355, BC5315, BC1735) were used to measure the amount of iron in cells/serum/tissues. Samples from cells/tissues were fully ground after adding the extraction solution, the supernatant was extracted after centrifugation, and the detection solution was added. The absorption peak was detected at 520 nm, and the iron content in the cells/tissues was obtained by comparison with the standard curve.

### Iron-induced and ovariectomy (OVX)-induced osteoporosis mouse model

For iron-induced osteoporosis mice, we used a modified version of Tsay’s method to construct a mouse model [11]. All animal experiments were approved by the Ethics Committee of Soochow University, approval No. SUDA20220925A03. Briefly, 6-week-old C57BL/6 male mice were assigned to the control, FAC, NC + FAC and sh-*Repin1* + FAC groups randomly. After two days of adaptive feeding, the FAC, NC + FAC and sh-*Repin1* + FAC groups received intraperitoneal injections of FAC (40 mg/kg/3 day) for 8 weeks, and the control mice received a same volume of saline instead. For OVX-induced osteoporosis mice, 6-week-old C57BL/6 female mice were assigned to the sham, OVX, NC + OVX and sh-*Repin1* + OVX groups randomly. After two days of adaptive feeding, only part of the fatty tissue around the ovary was removed in the sham group, while the OVX, NC + OVX and sh-*Repin1* + OVX groups received bilateral OVX operations. The lentivirus (in a volume of 25 µl) was injected into the marrow cavity of mice from the sh-*Repin1* + FAC or sh-*Repin1* + OVX groups through the periosteum and cortex of the femur at the epiphyseal transition once every two weeks. After injection, pressure was applied to stop bleeding. Mice were anesthetized with Nembutal during the operations and lentivirus injection. All animals survived and were fed in a constant temperature and humidity environment. Finally, all mice were sacrificed after 8 weeks. At the time of execution, about 1.5 ml blood was collected by eyeball removal, and the serum was collected after centrifugation (3000 × *g*, 4 °C) for 30 min and frozen at −80 °C for future analysis. All femurs were isolated for micro-CT and histology experiments, and the tibias were collected for protein and RNA collection.

### Clinical specimen collection

Bone tissue was obtained from hospitalized patients undergoing total hip replacement, during which the excised femoral neck was selected as follow-up tissue. According to the bone mineral density of femoral neck before operation, the samples were divided into osteoporosis group and control group. The bone tissues were quickly frozen with liquid nitrogen and fully ground, then RIPA or TRIzol was added for subsequent western blotting or qRT‒PCR analysis. All experiments had been approved by the Ethics Committee of the First Affiliated Hospital of Soochow University, Approval No. 379, 2022.

### Micro-CT analysis

Femurs from different groups were scanned with a micro-CT (SkyScan 1176, Karlsruhe, Germany) (*n* = 6/group). Mimics Medical (Materialize, Leuven, Belgium) was used to construct three-dimensional images of bone trabeculae. NRecon software (SkyScan N.V., Karlsruhe, Germany) was used to analyze the parameters of the distal femoral metaphyseal trabecular bone, including the bone mineral density (BMD, g/cm^3^), bone volume (BV, mm^3^), bone volume per tissue volume (BV/TV, %), bone surface per bone volume (BS/BV, 1/mm), trabecular thickness (Tb. th, mm), trabecular separation (Tb. sp, mm) and trabecular number (Tb. N, 1/mm). We chose 100 sections below the growth plate as the starting point, and included another 150 sections (6 µm each) below for analysis per sample.

### Alizarin red S double labeling staining

Dynamic bone formation was evaluated by using alizarin red S (ARS, Sigma‒Aldrich, Missouri, USA) double labeling staining. Mice were intraperitoneally injected with ARS 10 days and 3 days before death. The femur was immobilized in formalin for 48 h after isolation, and the tissue was sectioned using a freezing microtome (Leica, Heidelberg, Germany). The double-label distance was observed under a fluorescence microscope.

### Hematoxylin and eosin staining

The femurs from different groups were fixed in 10% formalin for 48 h. After 1 month of decalcification in 10% EDTA, after gradient dehydration, transparency and paraffin embedding, the specimens were sliced into 6 μm thick sections. A hematoxylin and eosin staining kit (Beyotime, Shanghai, China, C0105S) was used for hematoxylin and eosin (H&E) staining according to the protocol. The sections were air dried, sealed with neutral resin and then observed under a microscope.

### Statistical analysis

The data are displayed as the means ± standard deviations (means ± SDs). GraphPad Prism 8.0 software (GraphPad software, California, USA) was used for statistical analysis and graph drawing. Student’s *t* test was performed to determine the significance of differences between two groups, and one-way ANOVA followed by Tukey’s test was used to determine multiple comparisons. Statistical Product and Service Solutions (SPSS 25.0, IBM Inc., New York, USA) was used for statistical analysis. Statistical significance was defined as *p* < 0.05. ImageJ was used for quantitative fluorescence analysis, in brief, the pictures in the same group of experiments were taken at the same exposure time and brightness, the gamma value was uniformly set at 0.45. After adjusting the threshold and removing the background, we calculated the mean fluorescence intensity to compare the difference.

### Supplementary information

supplementary document

Original Data File

## Data Availability

The data sets used and/or analyzed during the current study are available from the corresponding author on reasonable request.
