# Supplementary material for: REPIN1 regulates iron metabolism and osteoblast apoptosis in osteoporosis
Source: Cell Death Dis. 2023 Sep 25;14(9):631. doi: 10.1038/s41419-023-06160-w (PMC10519990; doi:10.1038/s41419-023-06160-w)
Supplement: Supplementary file 1 — supplementary document [file 41419_2023_6160_MOESM1_ESM.docx]

**supplementary figures**


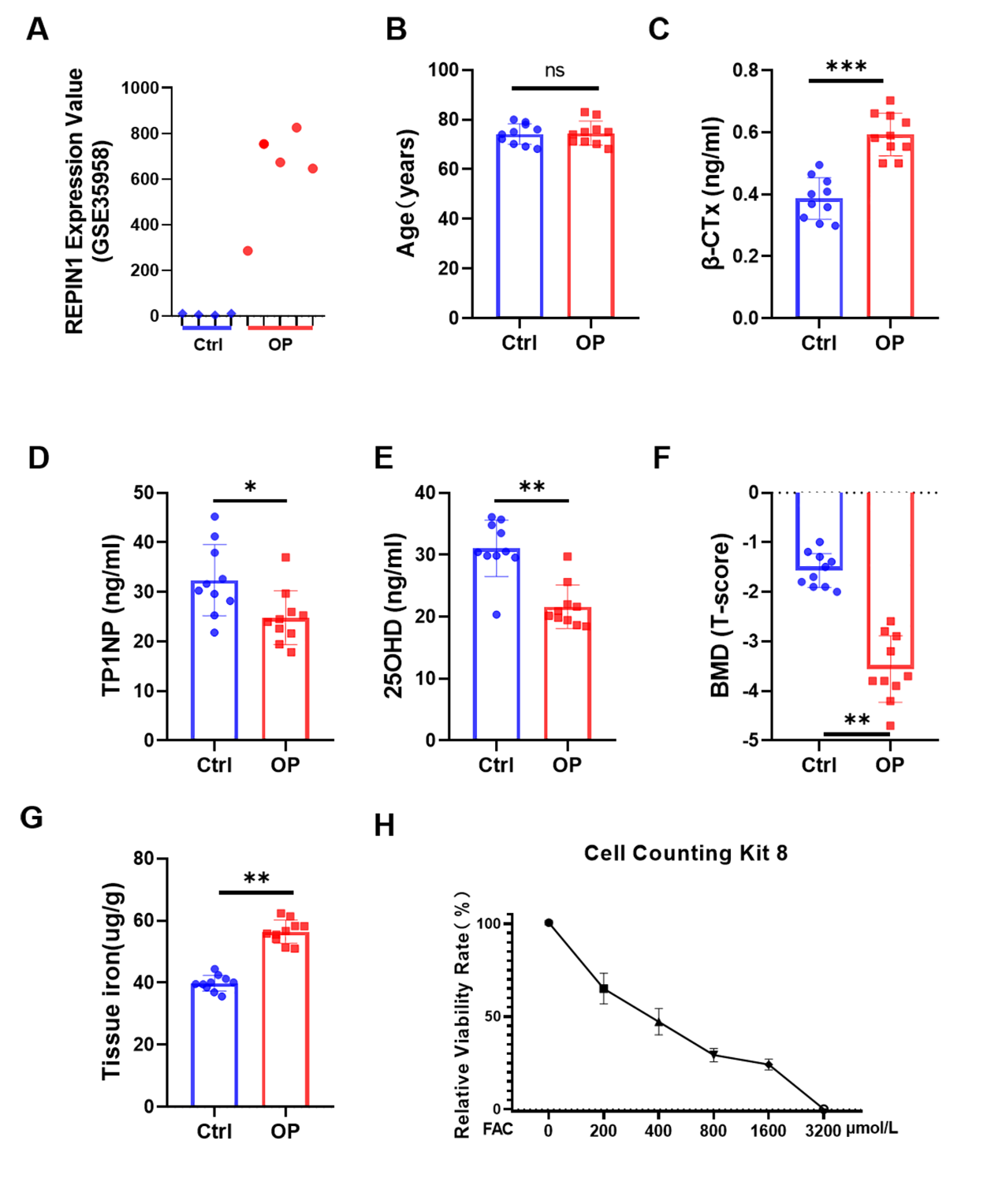


Figure S1. Iron overload exists in osteoporosis patients and shows dose-dependent cytotoxicity *in vitro*. (A) Expression of Repin1 in elderly versus elderly osteoporosis patients (data from GEO, GSE35958). (B-F) Bone metabolic parameters in osteoporosis patients. (B) Ages, (C) β-type I collagen carboxy-terminal peptide, β-CTx (ng/ml). (D) Total procollagen type 1 amino-terminal propeptide, TP1NP (ng/ml), (E) 25-hydroxy-vitamin D, 25 OHD (ng/ml), (F) Bone mineral density, BMD (T-score). (G) Iron content in bone tissues of osteoporosis patients (µg/g). (H) BMSCs were exposed to FAC at various concentrations (0-3200 μmol/L) for 48 h, and cell counting kit 8 (CCK8) was used to test cell viability. (Values are shown as the means ± SDs. **p* < 0.05, ***p* < 0.01 and ****p* < 0.005, n=10 per group.)


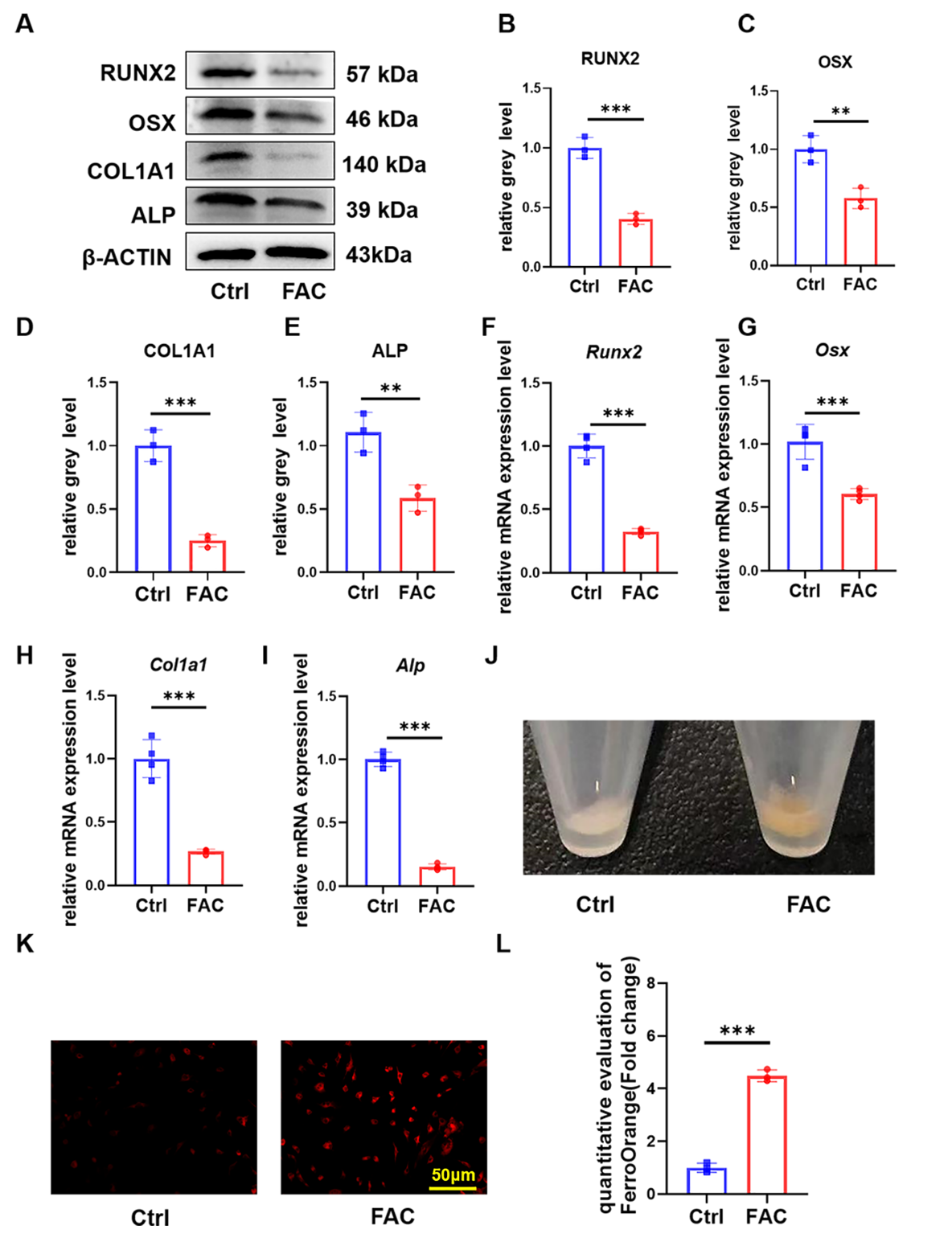


Figure S2. Iron overload impairs the osteogenic ability of BMSCs. (A-E) The expression levels of RUNX2, OSX, COL1A1 and ALP in BMSCs were analyzed by western blotting after 4 d of osteogenic induction in the presence of 200 μmol/L FAC. (F-I) The mRNA levels of *Runx2, Osx, Col1a1 and Alp* were analyzed by qRT‒PCR. (J) The appearance of BMSCs exposed to FAC. (K) FerroOrange fluorescent probe was used to measure intracellular iron content. (L) Quantitative evaluation of FerroOrange (Fold change). (Values are shown as the means ± SDs ***p* <0.01, ****p* < 0.005, n=3 per group, all studies were performed with at least three biological replicates.)


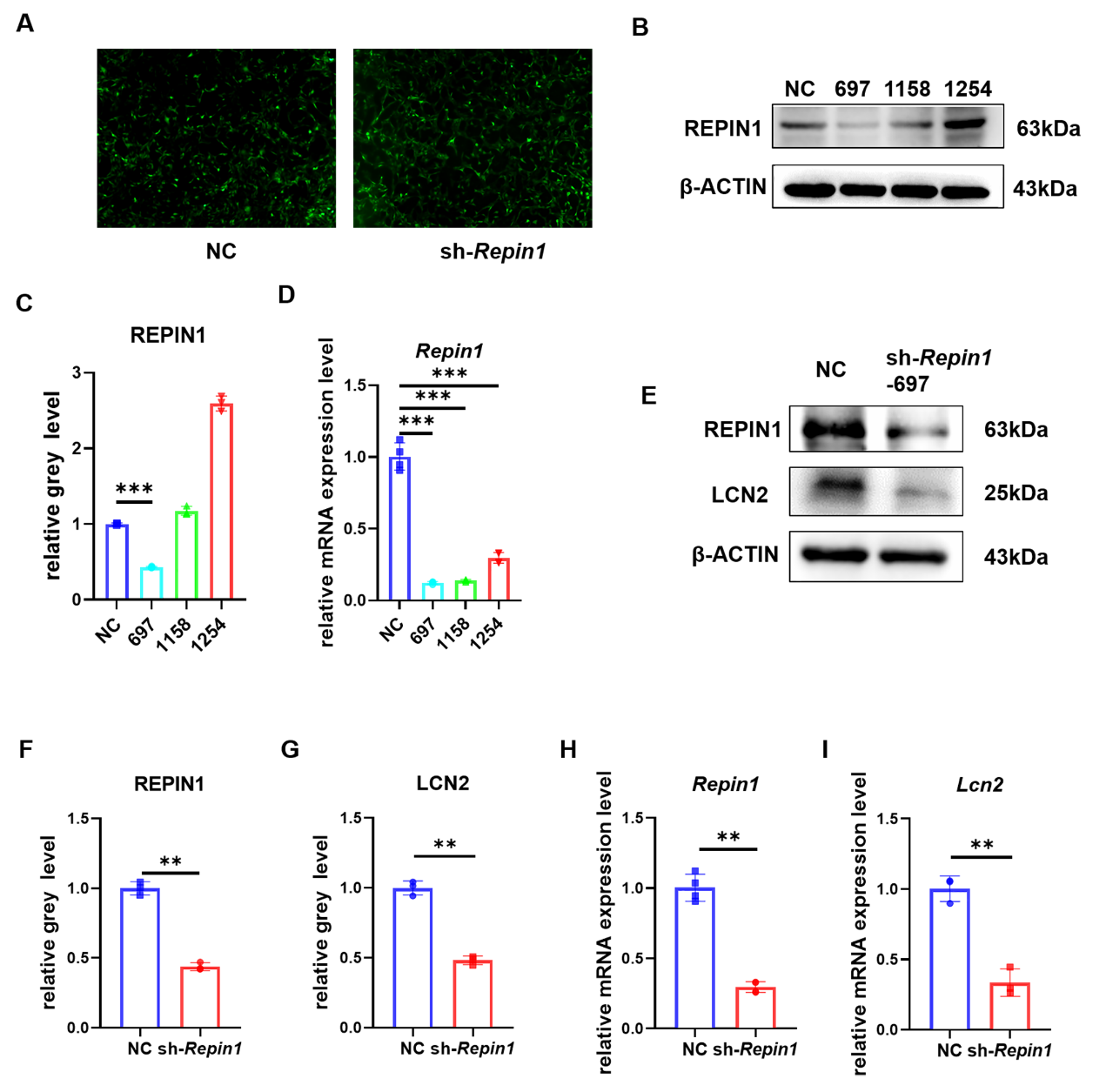


Figure S3. Lentivirus infectious efficiency in MC 3T3 E1 cells with NC and sh-*Repin1* (sh- *Repin1*-697, sh-*Repin1*-1158 and sh-*Repin1*-1254). (A) Representative GFP fluorescence images of MC 3T3 E1 cells 5 days after transfection with NC or sh- *Repin1* lentiviruses. (B,C) Protein levels of REPIN1 in MC 3T3 E1 cells after transfection with different lentiviruses. (D) Relative mRNA expression of *Repin1* after transfection with different lentiviruses. (E-G) Protein levels of REPIN1 and LCN2 in MC 3T3 E1 cells after transfection with sh-*Repin1*-697. (H, I) Relative mRNA expression of *Repin1* and *Lcn2* after transfection with sh-*Repin1*-697. (Values are shown as the means ± SDs ***p* < 0.01, ****p* < 0.005, n=3 per group, all studies were performed with at least three biological replicates.)


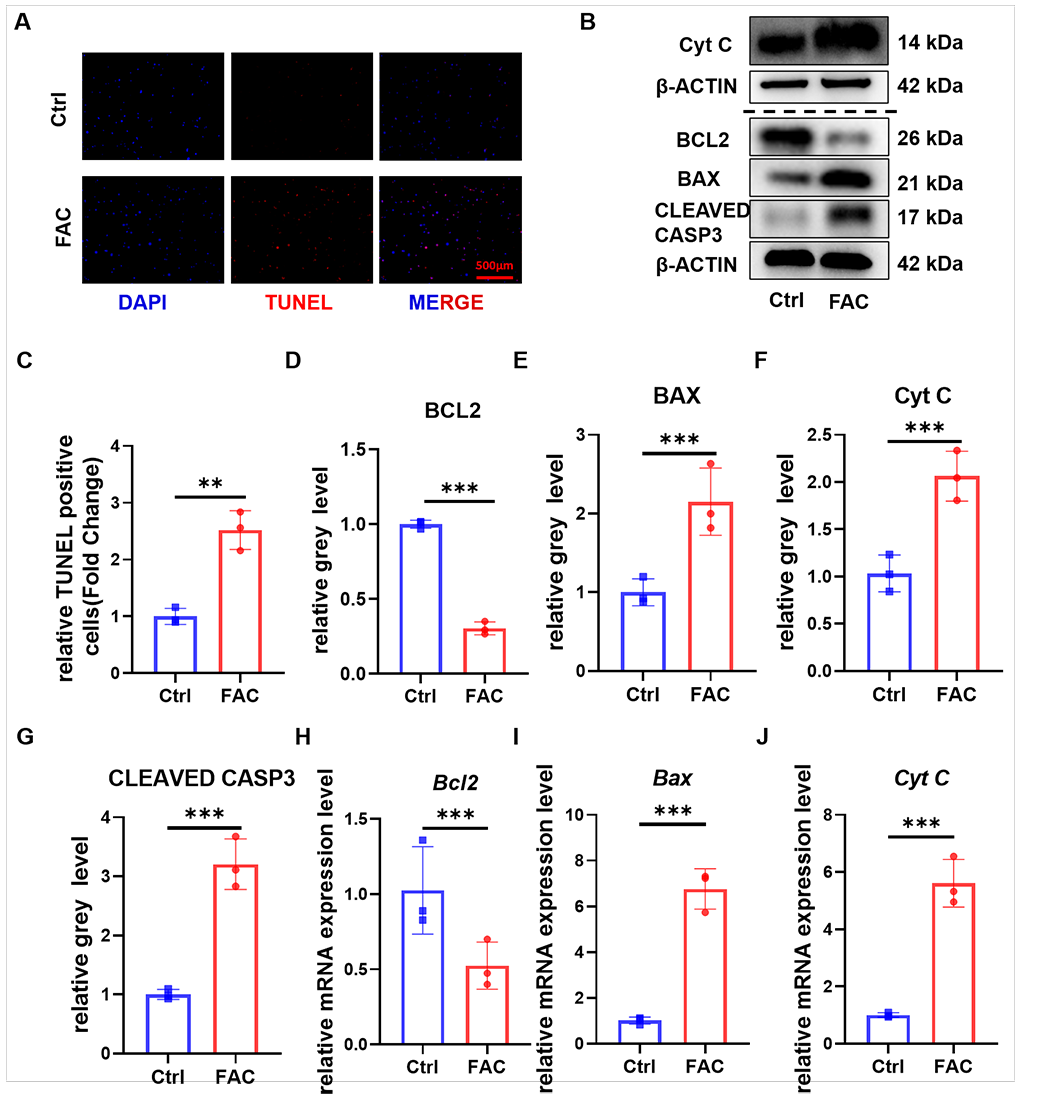


Figure S4. Iron overload induced apoptosis in BMSCs. (A) TUNEL staining of BMSCs. (B) Protein levels of Cyt C, BCL2, BAX, CLEAVED CASP3 in BMSCs. (C) Quantitative analysis of TUNEL-positive cells. (D-G) Quantitative analysis of western blotting. (H-J) Relative mRNA expression of *Cyt c, Bcl2* and *Bax* in BMSCs. (Values are shown as the means ± SDs **p < 0.01, ***p < 0.005, n=3 per group, all studies were performed with at least three biological replicates.)


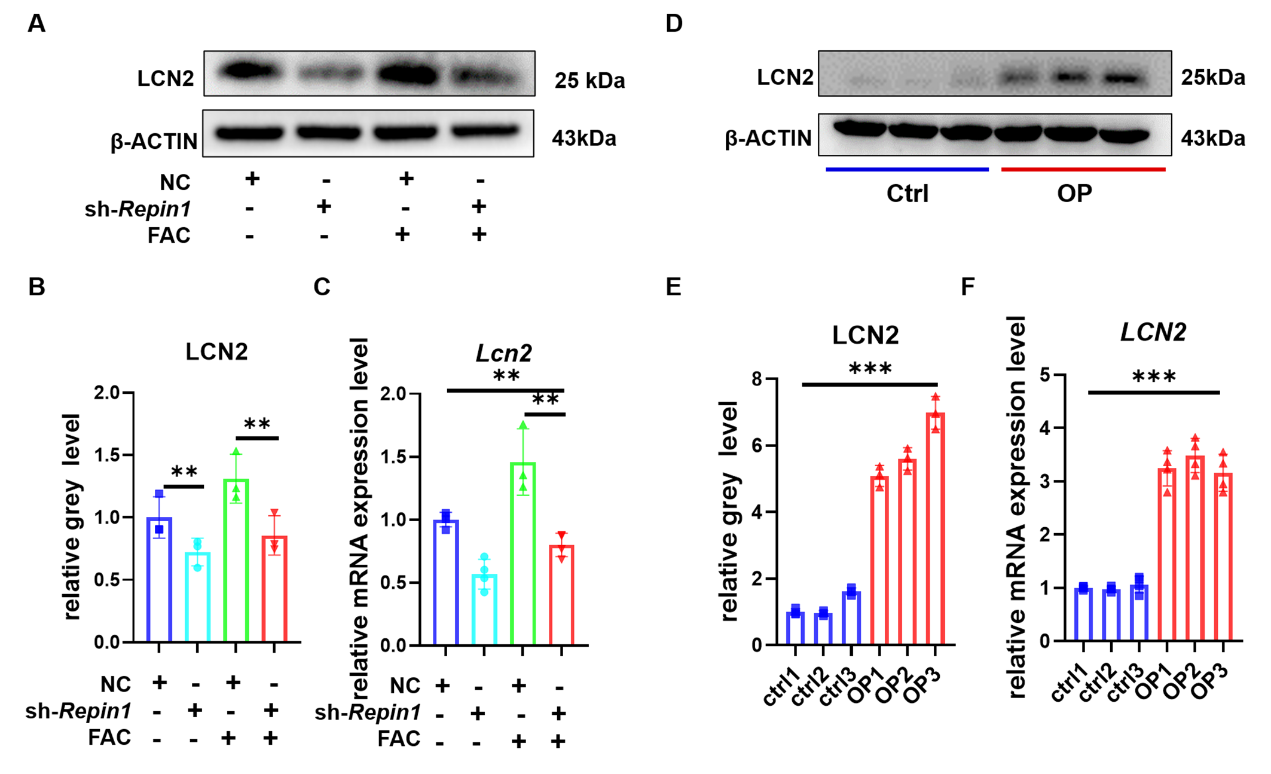


Figure S5. Expression level of LCN2 *in vivo* and *in vitro*. (A, B) The expression level of LCN2 in MC3T3 E1 cells was analyzed by western blotting. (C) Relative mRNA expression of *Lcn2*. (D, E) The expression level of LCN2 in patients was analyzed by western blotting. (F) Relative mRNA expression of *LCN2*. (Values are shown as the means ± SDs ****p* < 0.005, n=3 per group, all studies were performed with at least three biological replicates.)


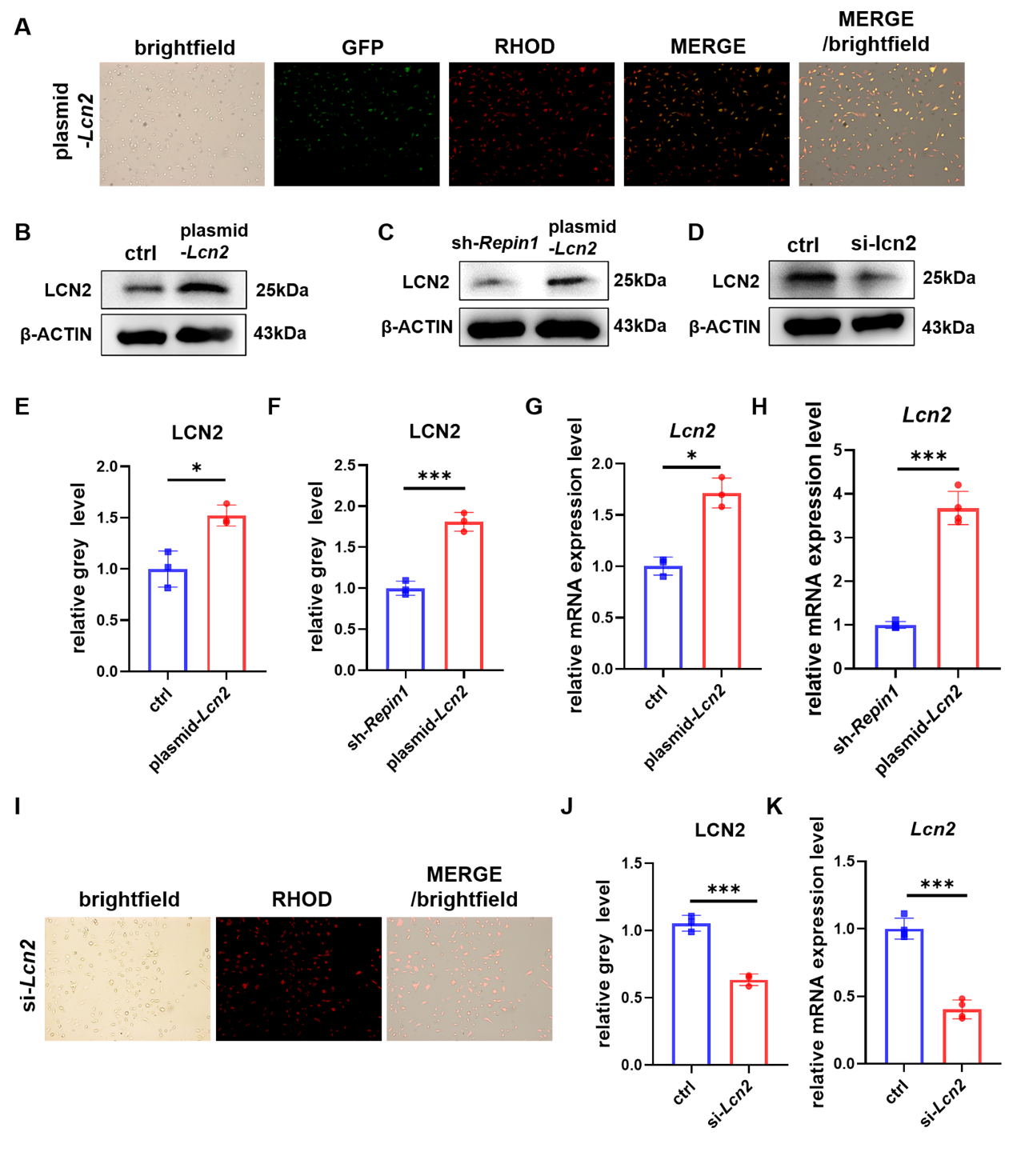


Figure S6. Infectious efficiency in MC 3T3 E1 cells with siRNA-*Lcn2* and plasmid-*Lcn2*. (A) Representative bright field images and GFP/RHOD fluorescence photographs of sh-*Repin1*-treated MC 3T3 E1 cells 8 h after transfection with plasmid-*Lcn2*. (B-D) The expression level of LCN2 was analyzed by western blotting. (E, F, J) Quantitative analysis of western blotting. (I) Representative bright field and GFP fluorescence images of MC 3T3 E1 cells 8 h after transfection with siRNA-*Lcn2*. (G, H, K) Relative mRNA expression of *Lcn2*. (Values are shown as the means ± SDs **p*<0.05, ****p* < 0.005, n=3 per group, all studies were performed with at least three biological replicates.)


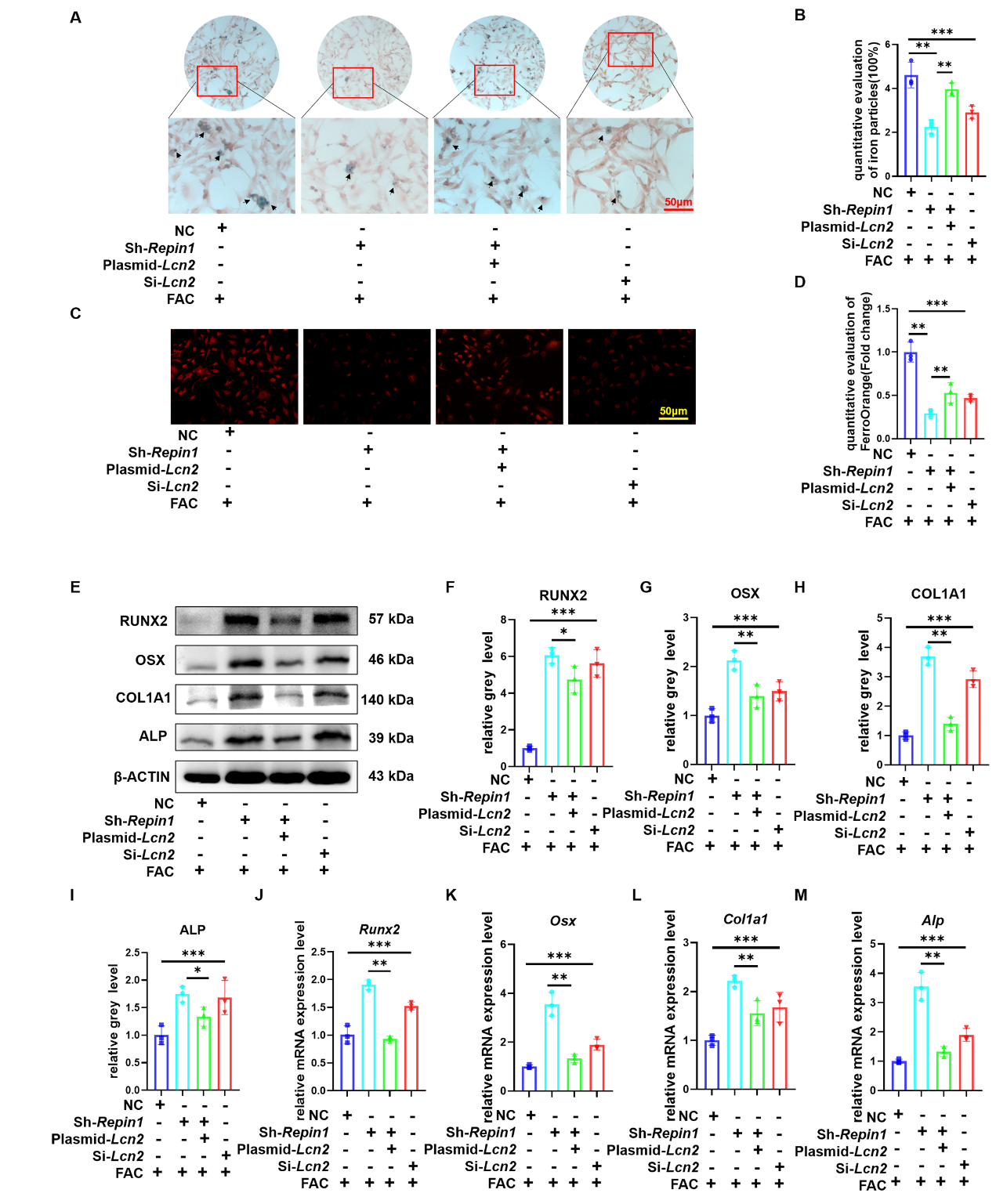


Figure S7. Altering the expression of *Lcn2* can reverse or mimic the effect of sh-*Repin1* on iron metabolism and osteogenic differentiation. (A) Perl’s Prussian blue staining was used to stain the iron particles in cells. (B) Semiquantitative evaluation of iron particles after Perl’s Prussian blue staining. (C) FerroOrange fluorescent probe was used to measure intracellular iron content. (D) Semiquantitative evaluation of FerroOrange. (E-I) The expression levels of RUNX2, OSX, COL1A1 and ALP were analyzed by western blotting. (J-M) Relative mRNA expression of *Runx2, Osx, Col1a1* and *Alp.* *(*Values are shown as the means ± SDs **p*<0.05, ***p* < 0.01, ****p* < 0.005, n=3 per group, all studies were performed with at least three biological replicates.)


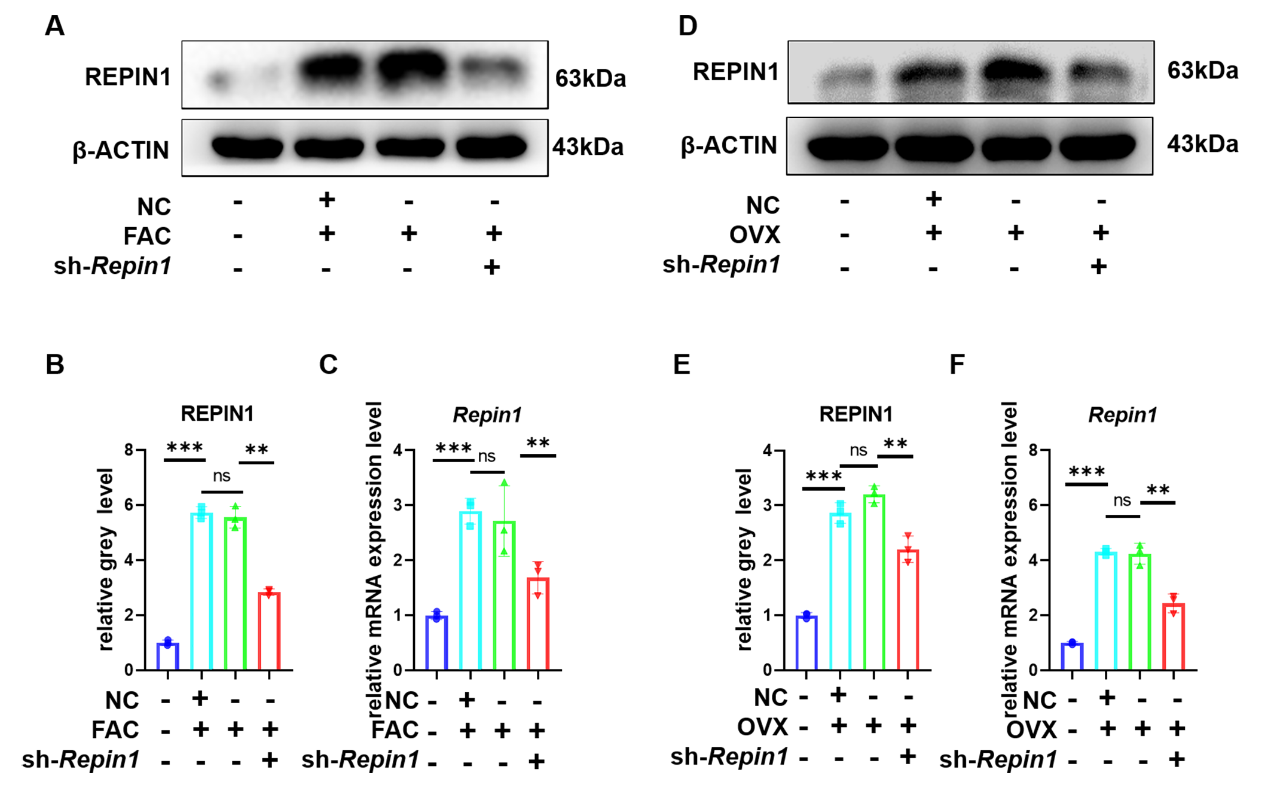


Figure S8. Expression level of REPIN1 in iron or OVX induced osteoporosis mouse models treated with *sh-Repin*1. (A) The expression level of REPIN1 in iron-induced osteoporosis mice was analyzed by western blotting. (B) Quantitative analysis of western blotting in (A). (C) Relative mRNA expression of *Repin1* in iron-induced osteoporosis mice. (D) The expression level of REPIN1 in OVX-induced osteoporosis mice was analyzed by western blotting. (E) Quantitative analysis of western blotting in (D). (F) Relative mRNA expression of *Repin1* in OVX-induced osteoporosis mice. (Values are shown as the means ± SDs ***p*<0.01, ****p* < 0.005, n=6 biologically independent mice per group)


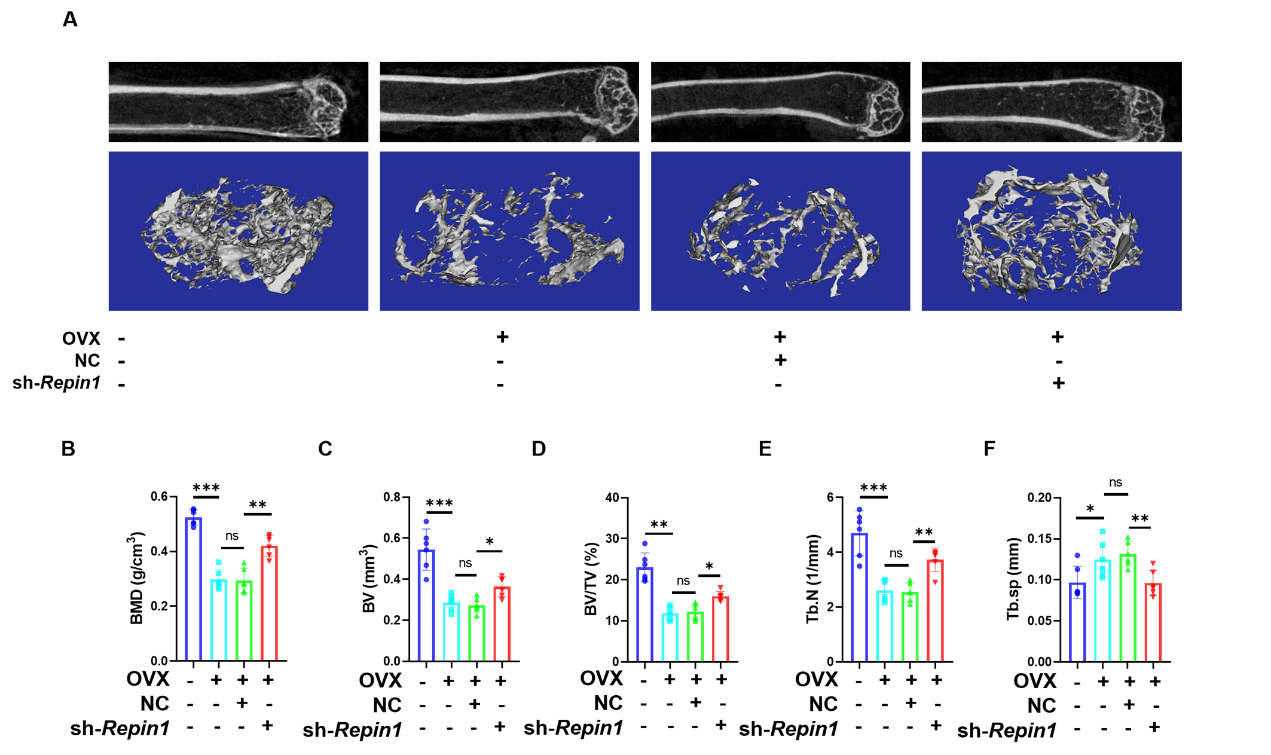


Figure S9. *Repin1* knockdown prevents OVX-induced bone loss *in vivo*. (A) Representative micro-CT reconstruction images of trabecular bone under the distal femur growth plate in the control group, OVX group, OVX+NC group, and OVX+sh-*Repin1* group are shown. (B-F) Quantitative analysis of bone parameters. The region of interest selected for trabecular analysis started 100 sections below the proximal end of the distal femur growth plate, and 150 slices (6 µm each) were read per sample. (B) BMD (g/cm^3^). (C) BV (mm^3^). (D) BV/TV (%). (E) Tb. N (1/mm). (F) Tb.sp (mm). (Values are shown as the means ± SDs. **p* < 0.05, ***p* < 0.01 and ****p* < 0.005, n=6 biologically independent mice per group.)


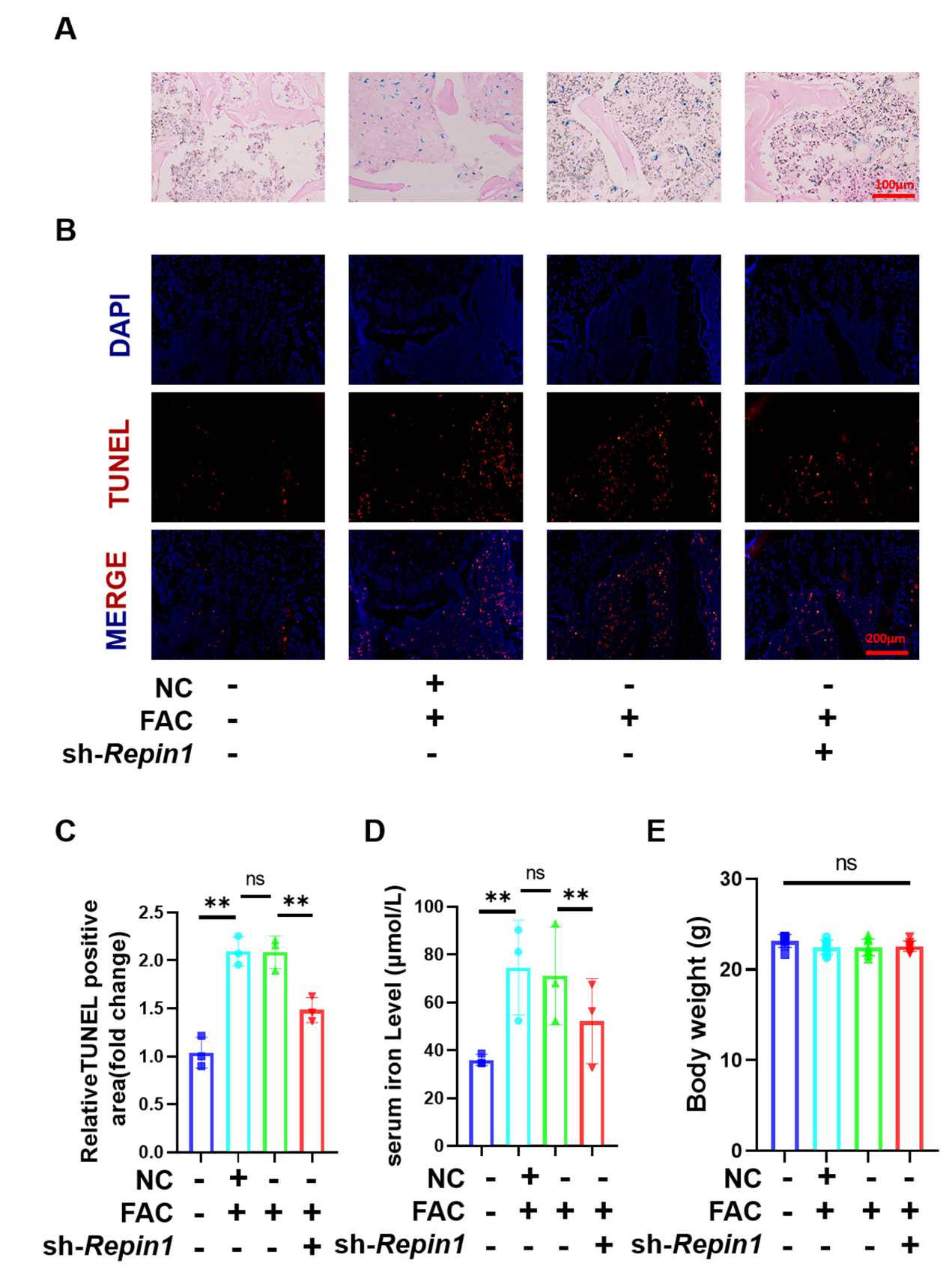


Figure S10. *Repin1* knockdown reduces iron particle deposition and iron-induced apoptosis *in vivo*. (A) Representative Perl’s Prussian blue staining images of trabecular bone under the distal femur growth plate from the control group, NC+FAC group, FAC group, and sh-*Repin1*+FAC group are shown. (B) TUNEL staining of bone tissues. (C) Quantitative analysis of the area of TUNEL-positive staining in (B). (D) Mouse serum iron levels. (E) Body weight of mice when sacrificed. (Values are shown as the means ± SDs. **p* < 0.05, ***p* < 0.01 and ****p* < 0.005, n=6 biologically independent mice per group)

Table S1 Primers of genes used in research

| Primer | 5'-3' |
| --- | --- |
| *Lcn2-F* | GAGTTACCCTGGATTAACGAGT |
| *Lcn2-R* | AAGCGGATGAAGTTCTCCTTTA |
| *Repin1-F* | GCAACAAGGCACGTCAGT |
| *Repin1-R* | GGAAGTGCCTTCGACAGT |
| *Gapdh-F* | GGTTGTCTCCTGCGACTTCA |
| *Gapdh-R* | TGGTCCAGGGTTTCTTACTCC |
| *Runx2-F* | CCTTCAAGGTTGTAGCCCTC |
| *Runx2-R* | GGAGTAGTTCTCATCATTCCCG |
| *Alp-F* | ATCTTTGGTCTGGCTCCCATG |
| *Alp-R* | TTTCCCGTTCACCGTCCAC |
| *Col1a1-F* | ACATGTTCAGCTTTGTGGACCT |
| *Col1a1-R* | GGACCCTTAGGCCATTGTGTA |
| *Osx-F* | AGGAGGCACAAAGAAGCCATACG |
| *Osx-R* | ATGCCTGCCTTGTACCACGAGC |
| *Cyt c-F* | ACAGCCAAGACAGTCGTTACACAG |
| *Cyt c-R* | AGACTTGTTGAGCGTGAAGCAGAC |
| *Bcl2-F* | GGTGGGGTCATGTGTGTGG |
| *Bcl2-R* | CGGTTC AGGTACTCAGTCATCC |
| *Bax-F* | TGAAGACAGGGGCCTTTTTG |
| *Bax-R* | AATTCGCCGGAGACACTCG |
