# Supplementary material for: REPIN1 regulates iron metabolism and osteoblast apoptosis in osteoporosis
Source: Cell Death Dis. 2023 Sep 25;14(9):631. doi: 10.1038/s41419-023-06160-w (PMC10519990; doi:10.1038/s41419-023-06160-w)
Supplement: Supplementary file 2 — Original Data File [file 41419_2023_6160_MOESM2_ESM.pptx]

## Slide 1
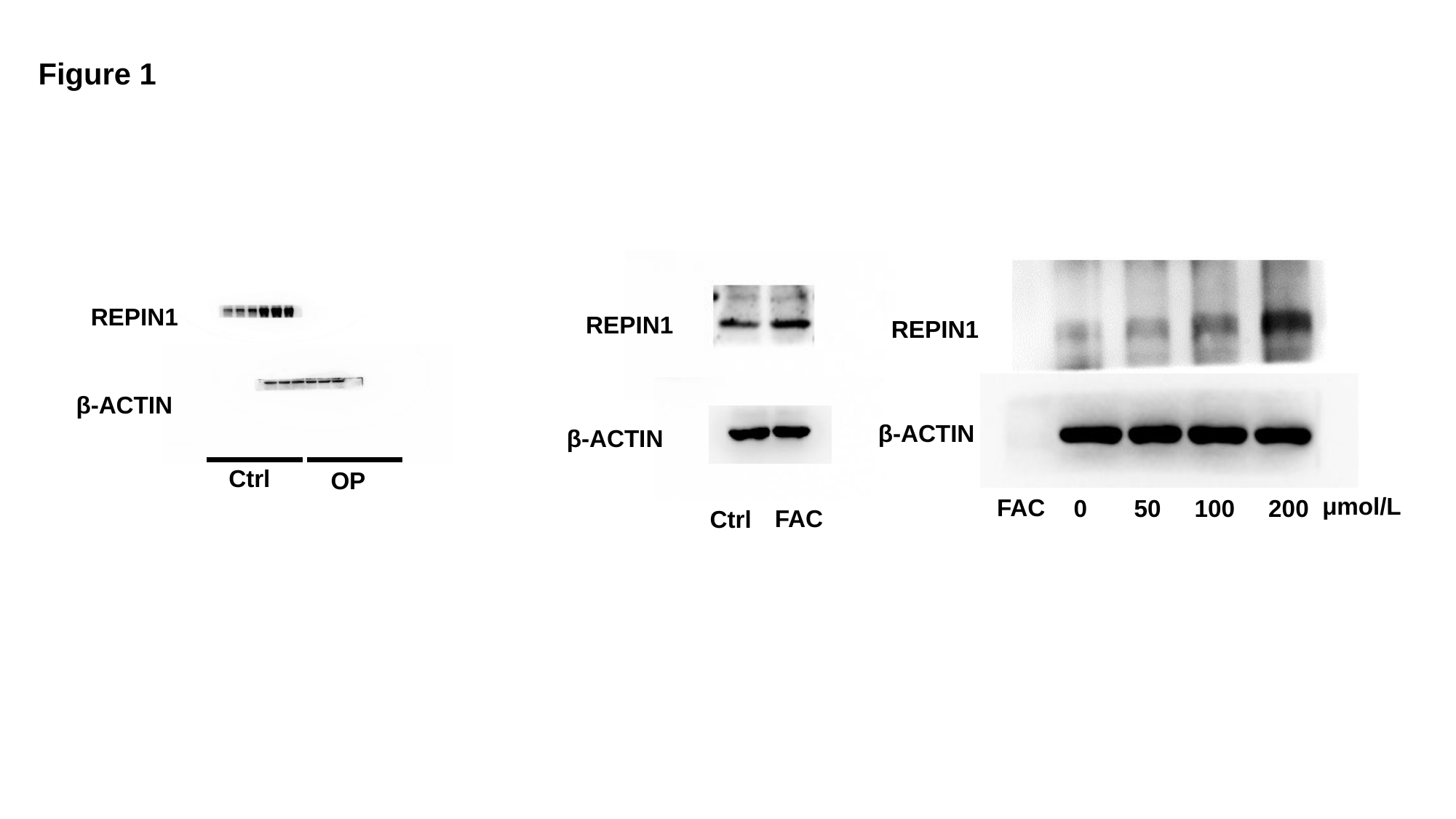

Figure 1
REPIN1
REPIN1
REPIN1
β-ACTIN
β-ACTIN
β-ACTIN
Ctrl
OP
μmol/L
FAC
0 50 100 200
FAC
Ctrl

## Slide 2
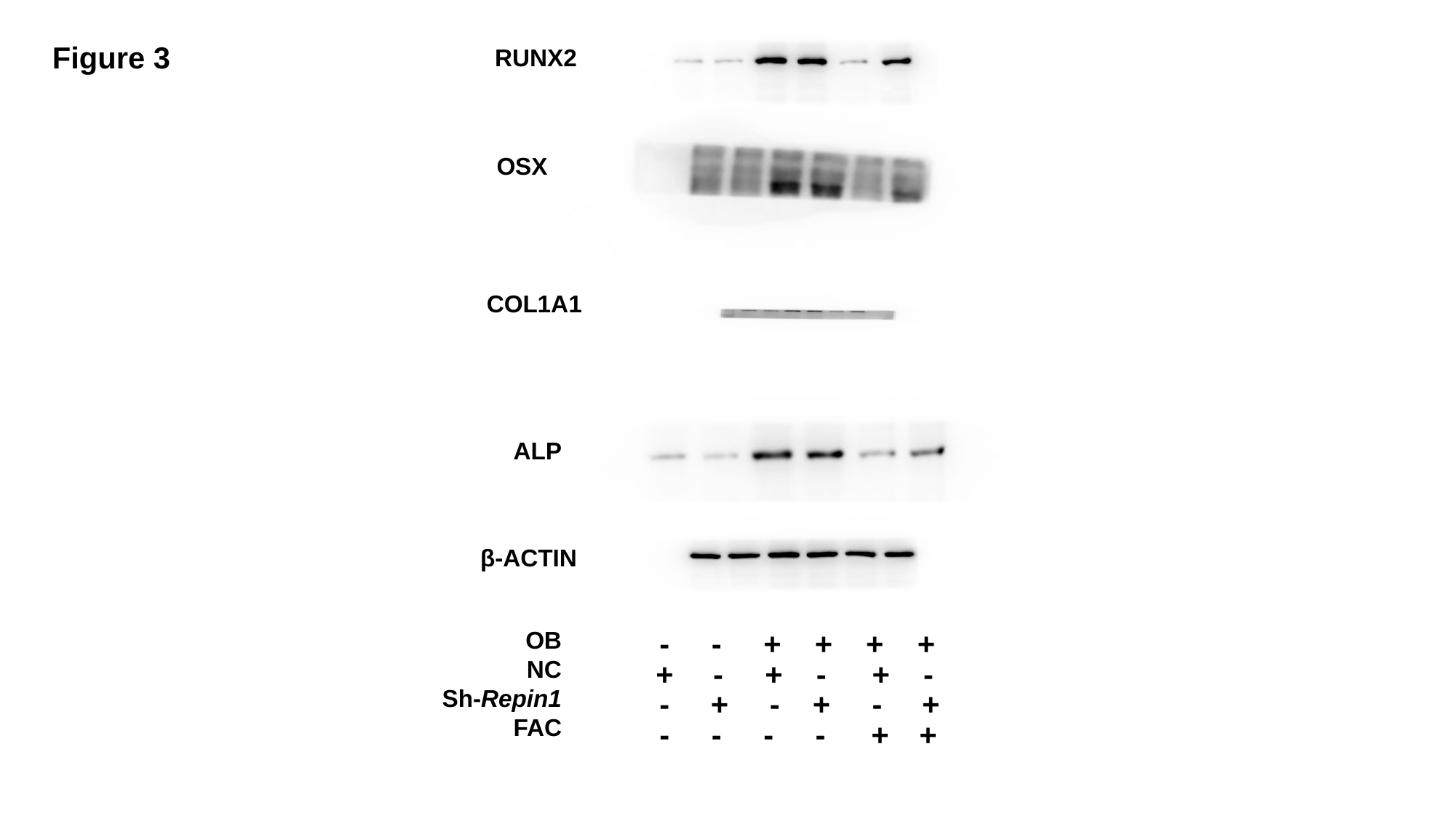

Figure 3
RUNX2
OSX
COL1A1
ALP
β-ACTIN
OB
NC
Sh-Repin1
FAC
 - - + + + +
 + - + - + -
 - + - + - +
 - - - - + +

## Slide 3
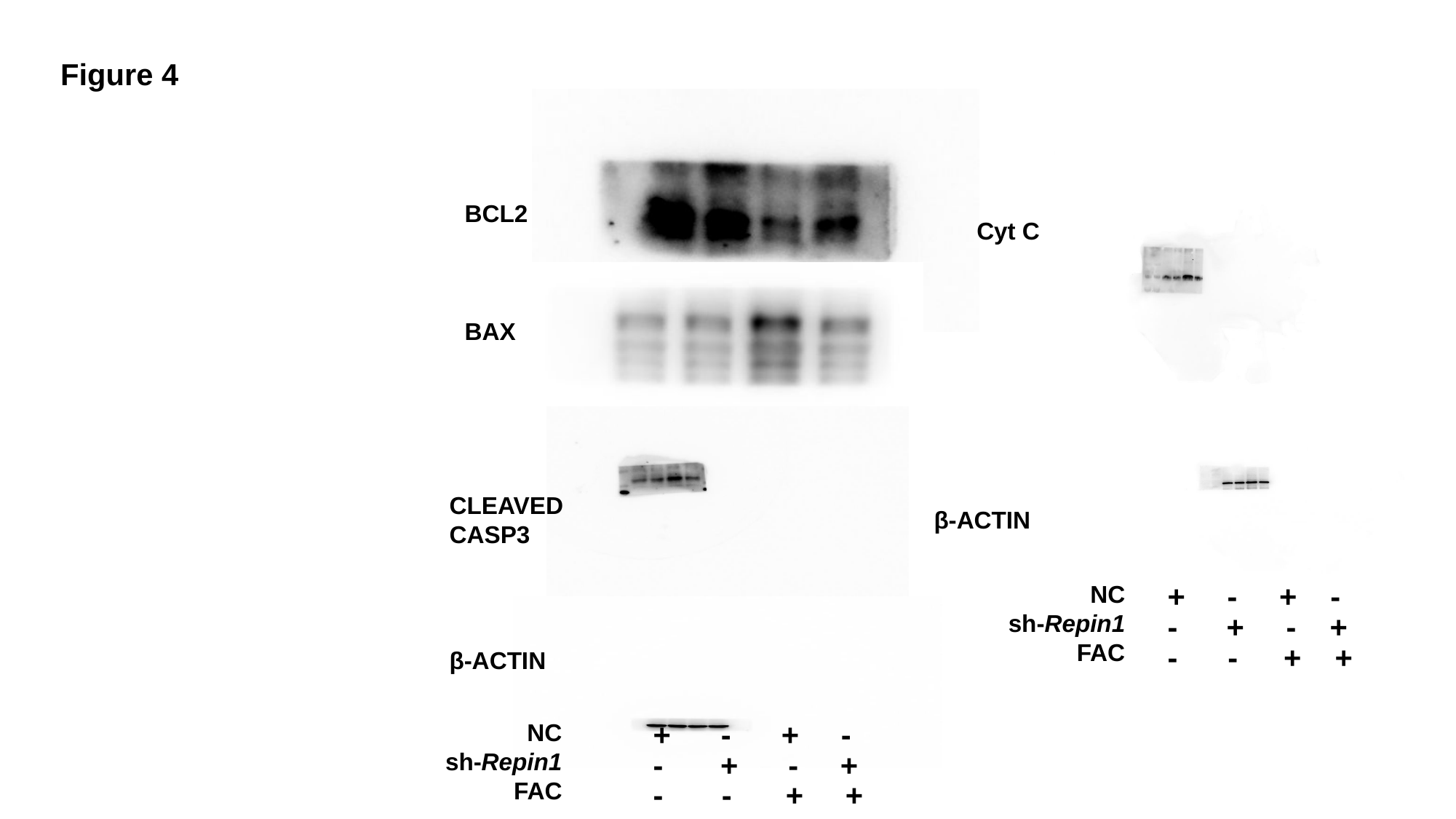

Figure 4
BCL2
Cyt C
BAX
CLEAVED
CASP3
β-ACTIN
NC
sh-Repin1
FAC
+ - + -
- + - +
- - + +
β-ACTIN
NC
sh-Repin1
FAC
+ - + -
- + - +
- - + +

## Slide 4
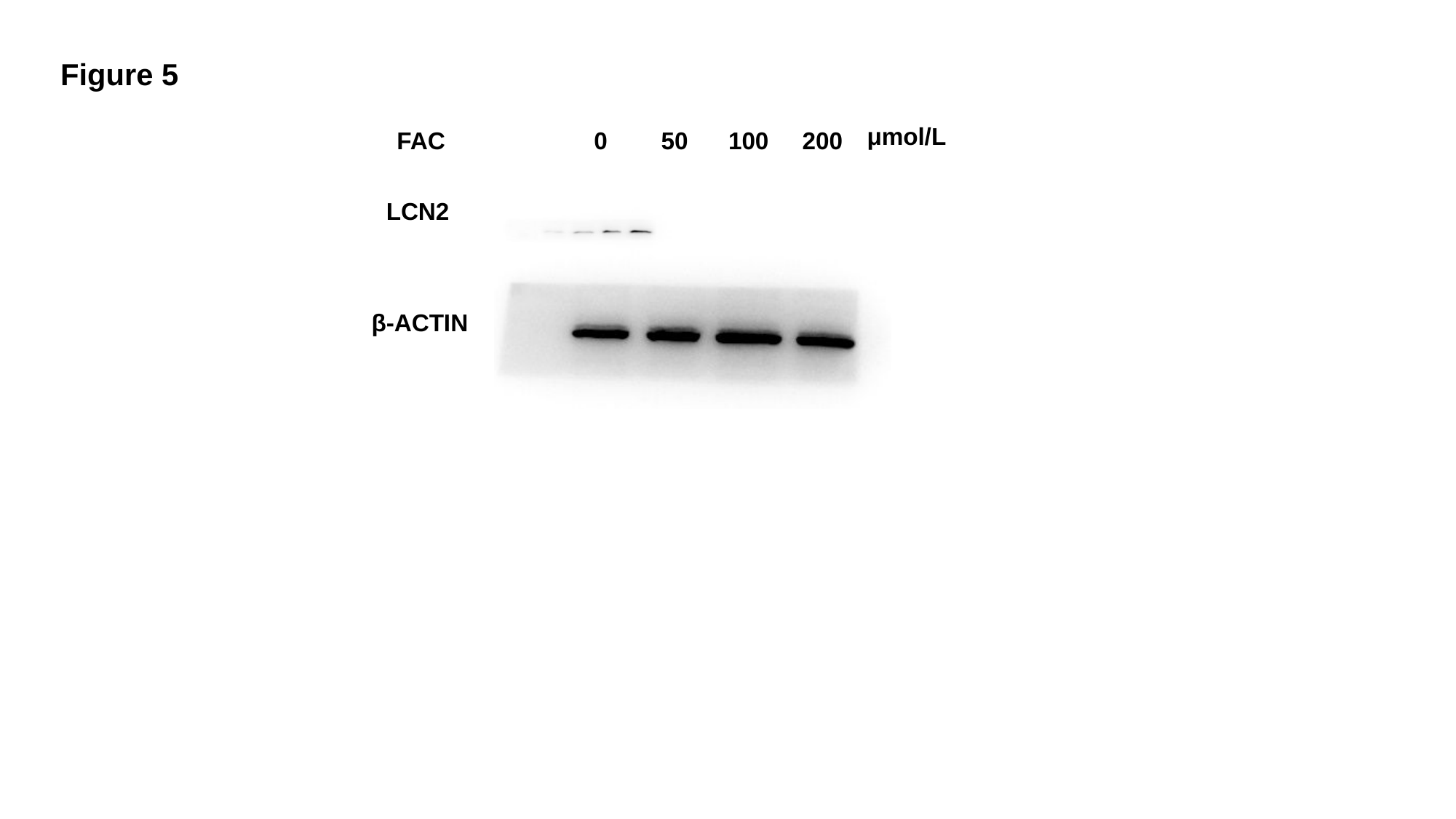

Figure 5
μmol/L
FAC
0 50 100 200
LCN2
β-ACTIN

## Slide 5
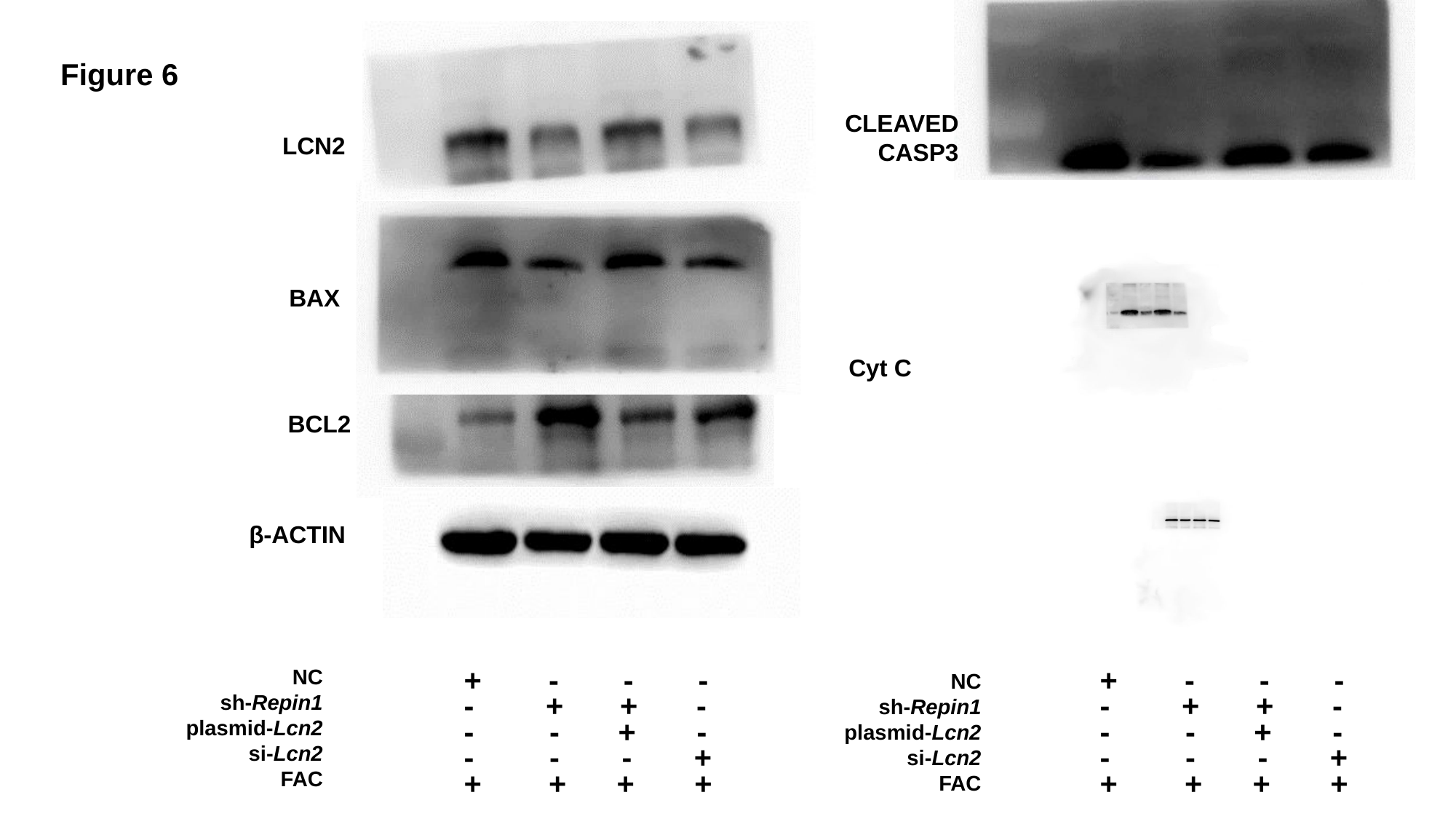

Figure 6
CLEAVED
CASP3
LCN2
BAX
Cyt C
BCL2
β-ACTIN
NC
sh-Repin1
plasmid-Lcn2
si-Lcn2
FAC
NC
sh-Repin1
plasmid-Lcn2
si-Lcn2
FAC
+ - - -
- + + -
- - + -
- - - +
+ + + +
+ - - -
- + + -
- - + -
- - - +
+ + + +

## Slide 6
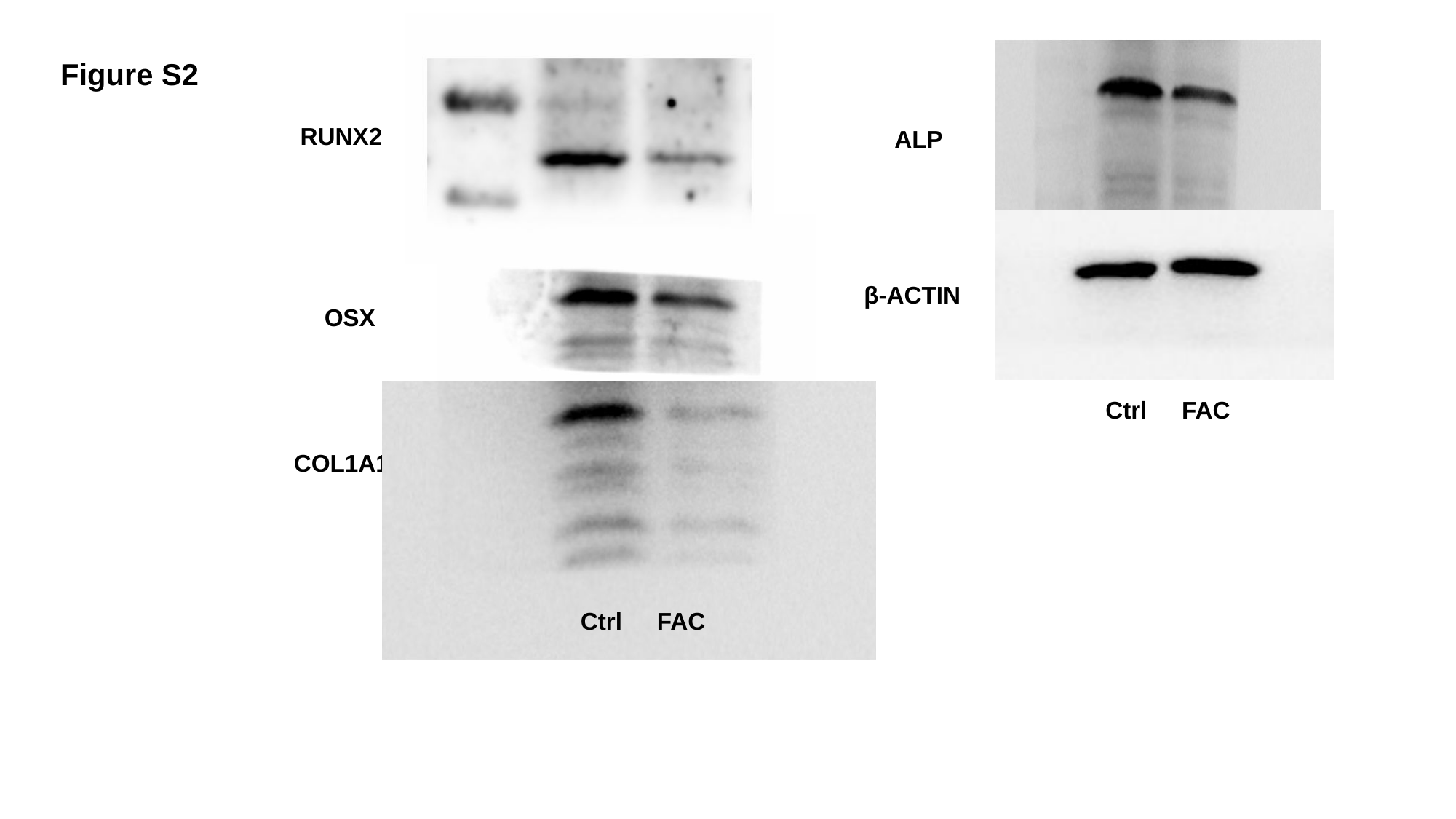

Figure S2
RUNX2
ALP
β-ACTIN
OSX
Ctrl
FAC
COL1A1
Ctrl
FAC

## Slide 7
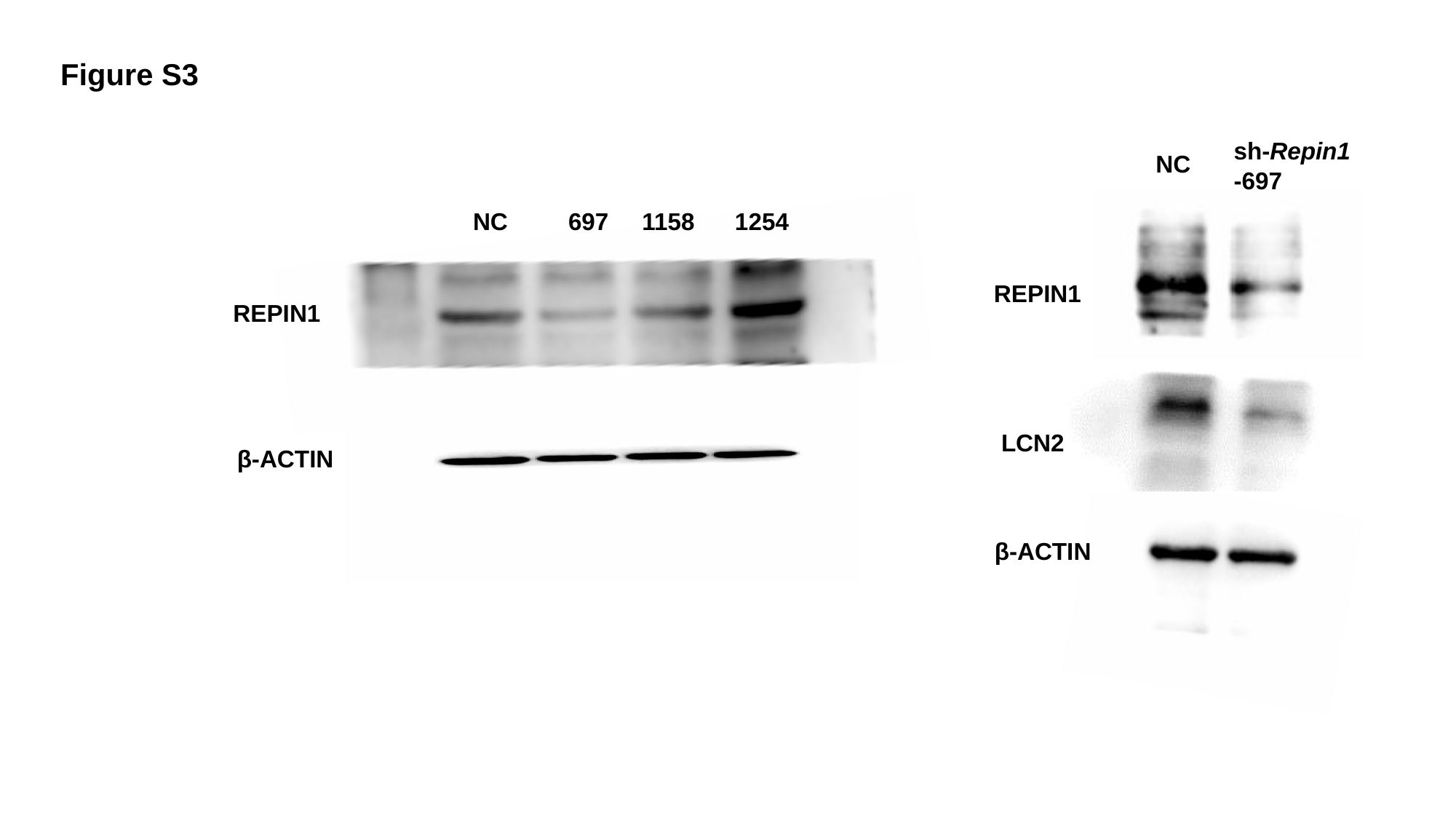

Figure S3
sh-Repin1
-697
NC
NC 697 1158 1254
REPIN1
REPIN1
LCN2
β-ACTIN
β-ACTIN

## Slide 8
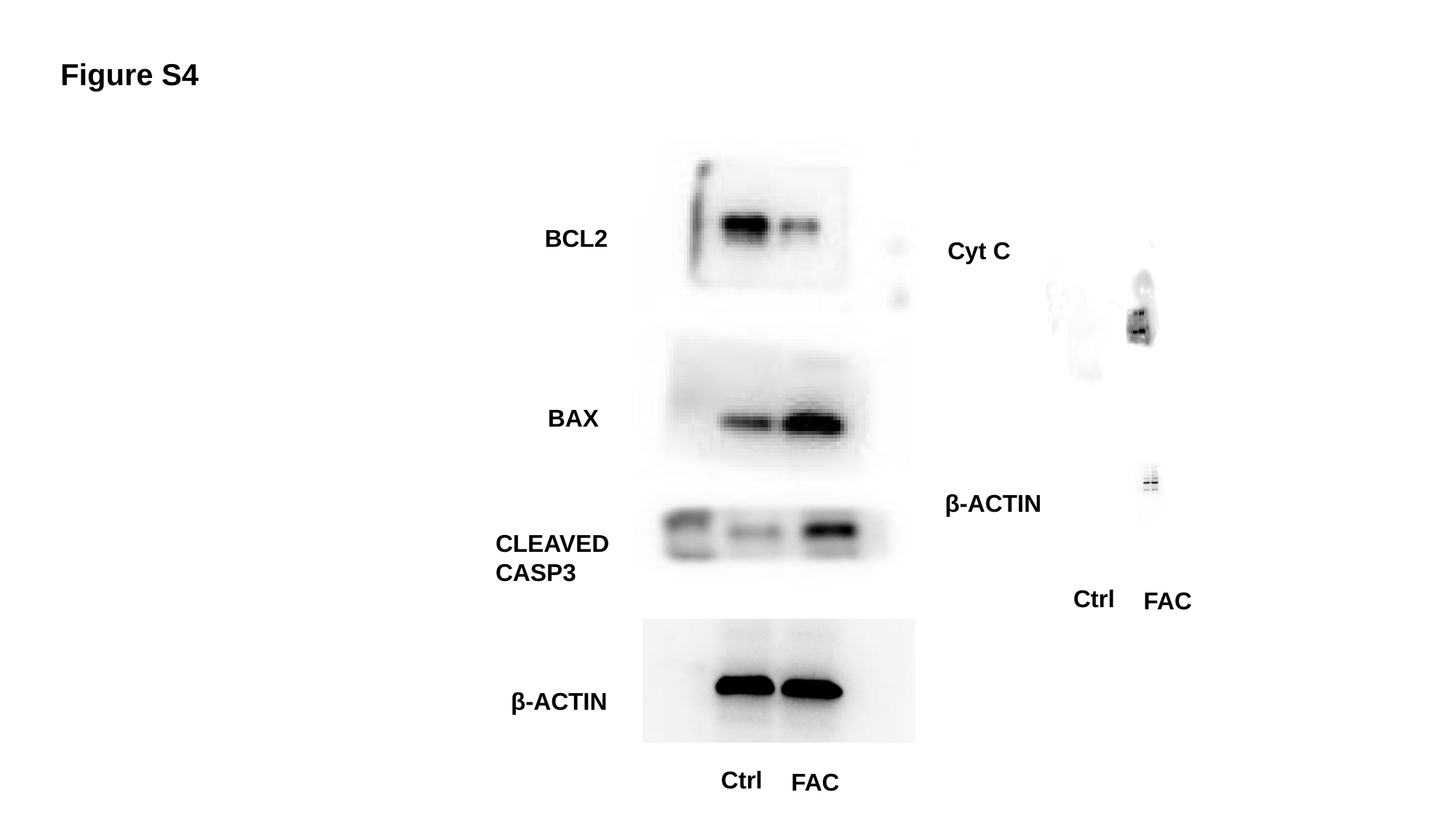

Figure S4
BCL2
Cyt C
BAX
β-ACTIN
CLEAVED
CASP3
Ctrl
FAC
β-ACTIN
Ctrl
FAC

## Slide 9
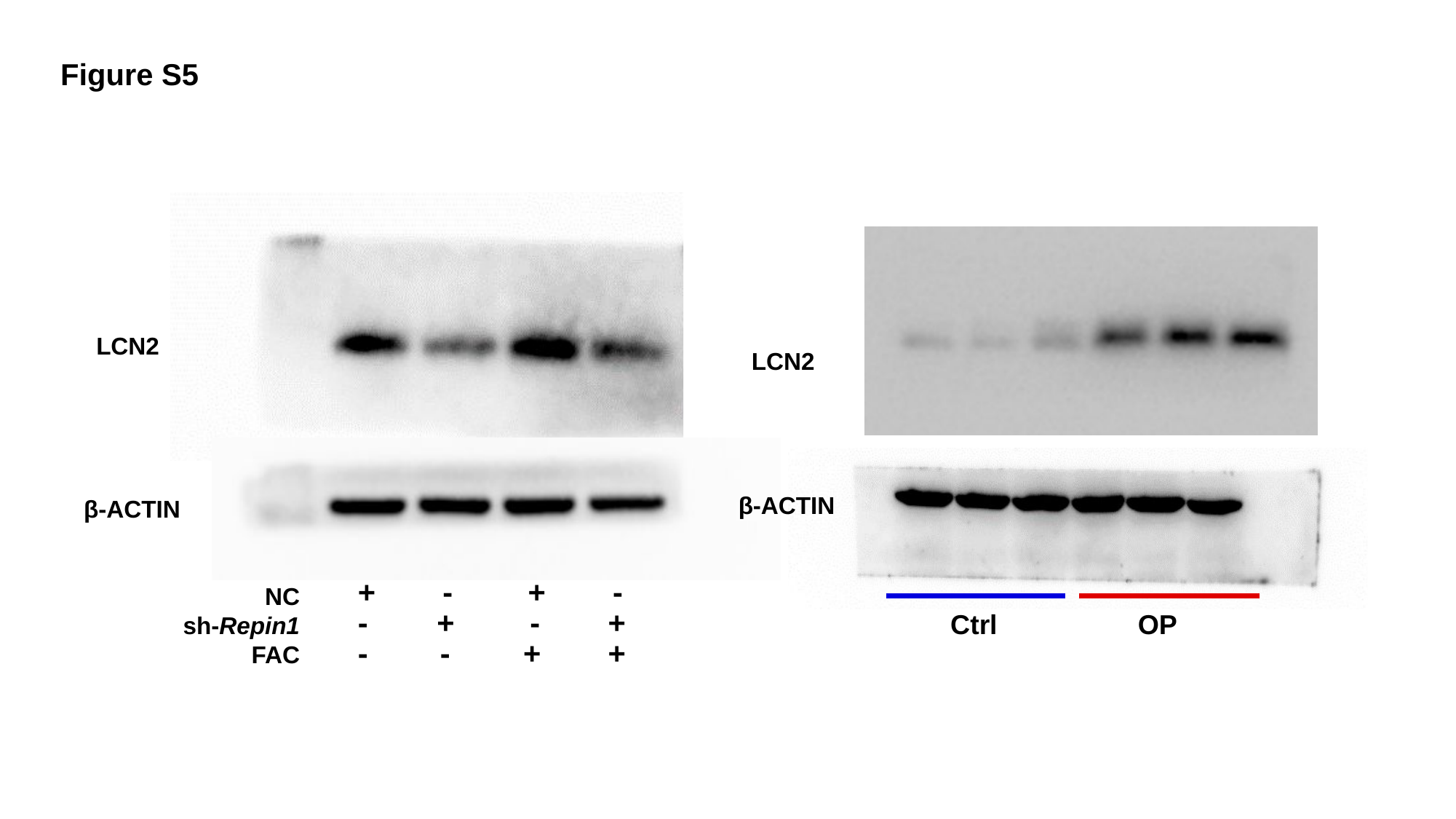

Figure S5
LCN2
LCN2
β-ACTIN
β-ACTIN
+ - + -
- + - +
- - + +
NC
sh-Repin1
FAC
Ctrl
OP

## Slide 10
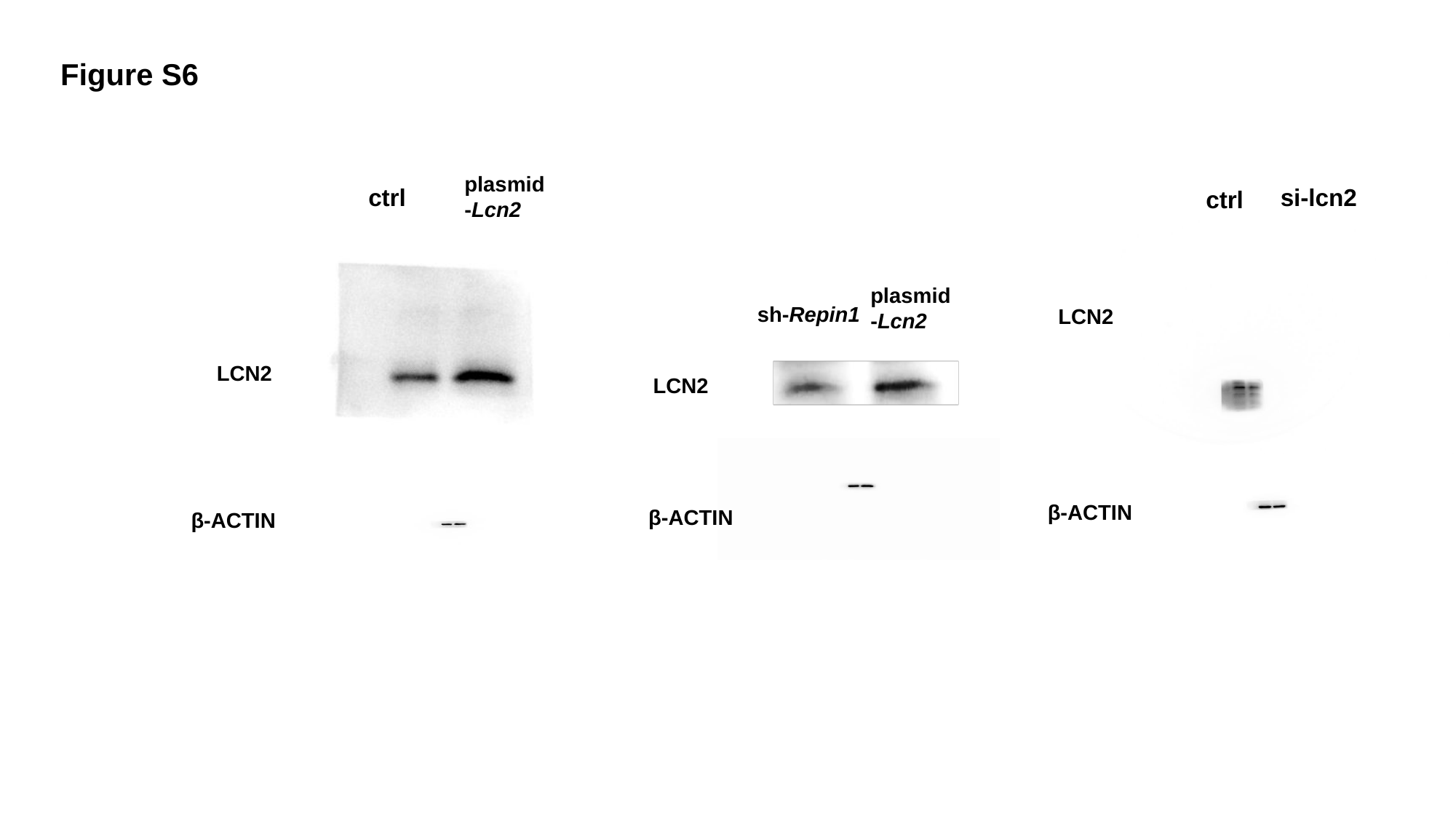

Figure S6
plasmid
-Lcn2
ctrl
si-lcn2
ctrl
plasmid
-Lcn2
sh-Repin1
LCN2
LCN2
LCN2
β-ACTIN
β-ACTIN
β-ACTIN

## Slide 11
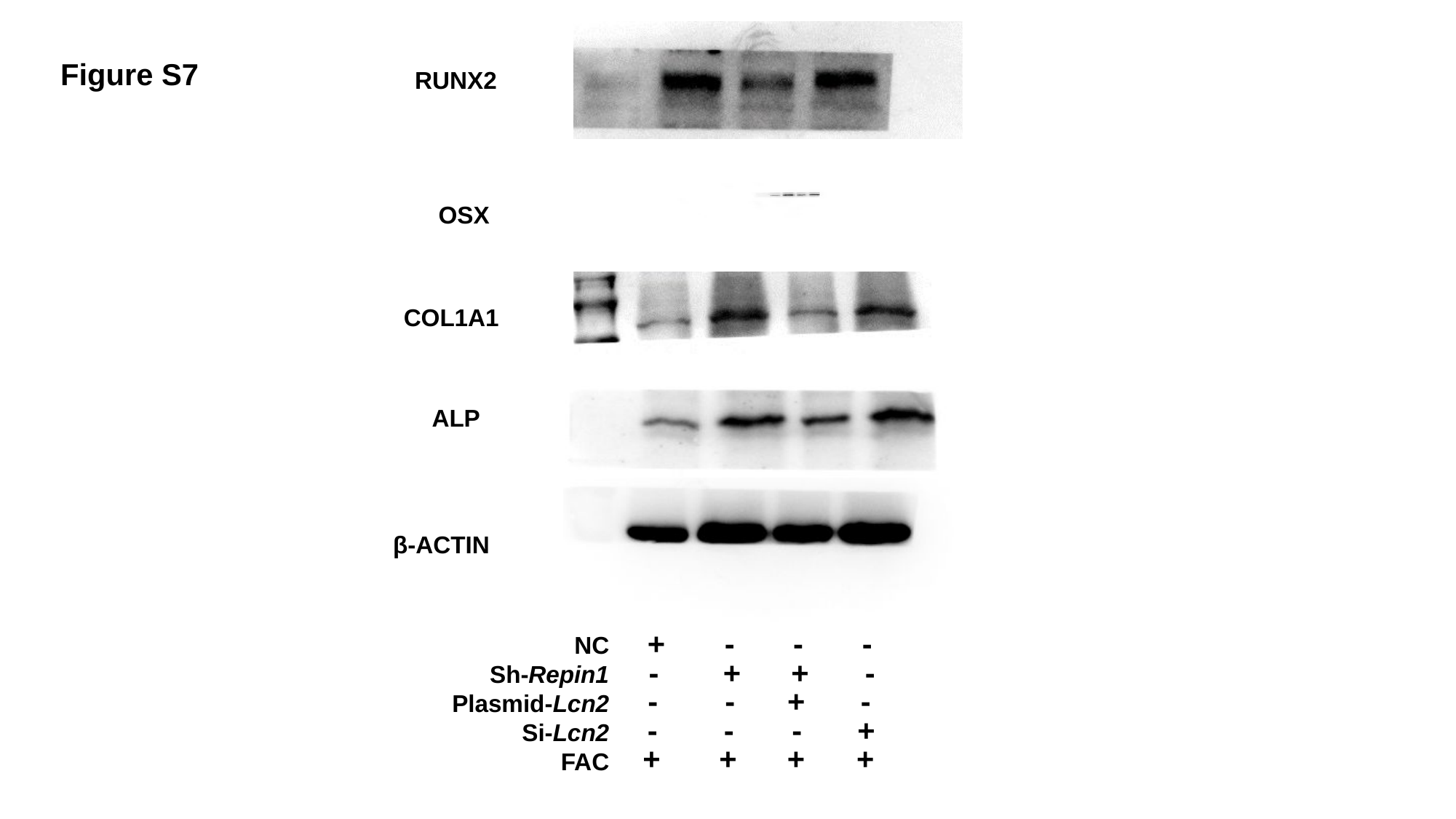

Figure S7
RUNX2
OSX
COL1A1
ALP
β-ACTIN
NC
Sh-Repin1
Plasmid-Lcn2
Si-Lcn2
FAC
 + - - -
 - + + -
 - - + -
 - - - +
+ + + +

## Slide 12
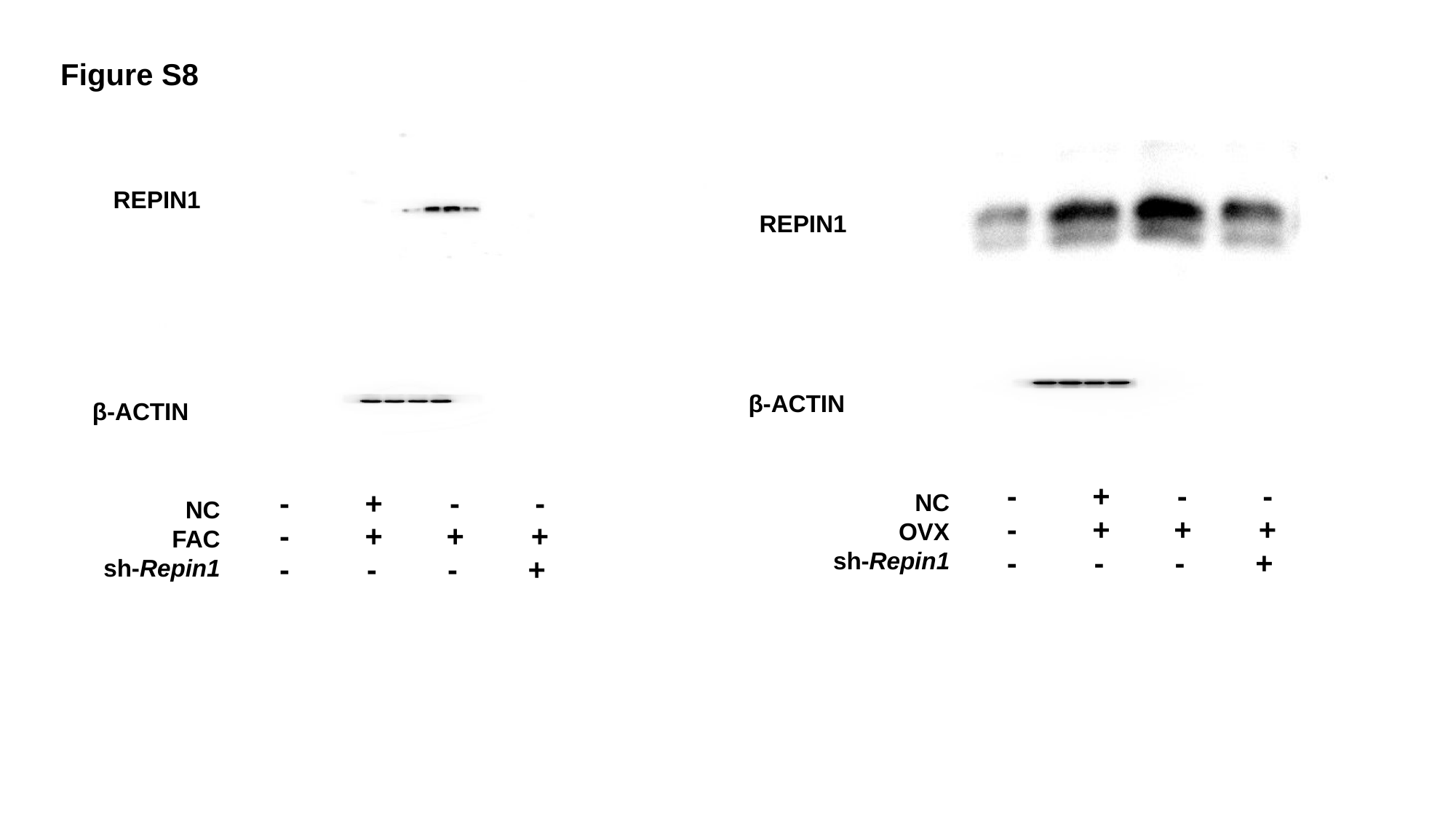

Figure S8
REPIN1
REPIN1
β-ACTIN
β-ACTIN
- + - -
- + + +
- - - +
- + - -
- + + +
- - - +
NC
OVX
sh-Repin1
NC
FAC
sh-Repin1
